# DEPP: A diabetes exercise prescription protocol knowledge base

**DOI:** 10.1371/journal.pdig.0001728

**Published:** 2026-09-16

**Authors:** Ting Bao, Xingyun Liu, Shumin Ren, Ke Zhang, Jiale Du, Danting Li, Bingqing Liu, Jinhua Feng, Rongrong Wu, Erman Wu, Chaoying Zhan, Min Jiang, Li Shen, Cheng Bi, Yingbo Zhang, Sheyu Li, Juan M. Ruso, Bairong Shen

**Affiliations:** 1 Health Management Center, General Practice Medical Center and Institutes for Systems Genetics, Frontiers Science Center for Disease-related Molecular Network, West China Hospital, Sichuan University, Chengdu, China; 2 Department of Computer Science and Information Technology, University of A Coruña, Coruña, Spain; 3 Health Management Center, General Practice Medical Center, West China Hospital, Sichuan University, Chengdu, China; 4 Department of Epidemiology and Health Statistics, West China School of Public Health, Sichuan University, Chengdu, China; 5 Department of Biliary Surgery, West China Hospital of Sichuan University/West China School of Nursing, Sichuan University, Chengdu, China; 6 Institute for Molecular Medicine Finland (FIMM), HiLIFE, University of Helsinki, Helsink, Finland; 7 Department of Endocrinology and Metabolism, Division of Guideline and Rapid Recommendation, Cochrane China Centre, MAGIC China Centre, Chinese Evidence-Based Medicine Centre, West China Hospital, Sichuan University, Chengdu, China; 8 Soft Matter and Molecular Biophysics Group, Department of Applied Physics and Institute of Materials (iMATUS), University of Santiago de Compostela, Santiago de Compostela, Spain; Shanghai United Imaging Intelligence Co Ltd, CHINA

## Abstract

Exercise plays a critical role in diabetes management by improving glycemic control and reducing cardiovascular risk. However, personalizing exercise prescriptions to individual characteristics and preferences is challenging, especially given the global shortage of exercise professionals, and current approaches often rely on general guidelines that lack support for individualized, evidence-based planning. To address this gap, we developed the Diabetes Exercise Prescription Protocol (DEPP) knowledge base, a structured evidence resource to assist healthcare experts in formulating personalized exercise prescriptions. DEPP was constructed by manually extracting and annotating data from 529 English-language studies retrieved from PubMed, resulting in a curated set of 766 exercise prescription protocols. The system uses a browser/server architecture implemented with Flask, SQLite, and Waitress. The knowledge base enables personalized retrieval and comparison of evidence-based protocols using a novel “FITT-VP-WC” framework, which extends the established FITT-VP principles by explicitly integrating warnings and contraindications as core safety components. Usability and clinical utility were evaluated with 12 sports physicians and 16 students (SUS/NPS) and in a vignette-based comparison with 20 clinicians (DEPP vs. no tool). The overall SUS score was 80.18 (physicians: 83.13, students: 77.97, p > 0.05) and NPS was 28.6% (physicians: 50.0%, students: 12.5%, p > 0.05), indicating good acceptance. In the comparative study, DEPP-assisted prescriptions significantly outperformed those without the tool across three standardized patient cases (all p < 0.001), confirming its practical benefit. The knowledge base is freely accessible at http://depp.sysbio.org.cn. This resource offers a new tool for evidence-based, personalized exercise prescription in diabetes care with positive user feedback.

## Author summary

Regular physical activity is just as critical as medication or diet for managing diabetes, yet many patients struggle to get safe, personalized exercise guidance. The reality is that most clinical guidelines offer one-size-fits-all advice, and access to trained exercise specialists is limited. This leaves healthcare providers with little support when tailoring physical activity plans for individuals with varying complications, fitness levels, and health risks. To tackle this challenge, we built the Diabetes Exercise Prescription Protocol (DEPP), a free, online knowledge base designed to put evidence-based exercise recommendations directly into clinicians’ hands. Our team analyzed 529 scientific studies published over nearly four decades (1986–2025) and curated 766 specific exercise protocols. Unlike standard guides, DEPP introduces a practical framework that not only covers exercise type, intensity, and duration, but also highlights critical safety warnings and contraindications, helping doctors avoid potentially dangerous advice. We tested DEPP with real users, including sports physicians and medical students—and found that it scored 80.18 out of 100 on the System Usability Scale, placing it in the “excellent” range for ease of use. Its Net Promoter Score of 28.6% further indicated that users would generally recommend it to peers. More importantly, when we asked 20 clinicians to prescribe exercise plans both with and without DEPP, the protocols generated using our platform were significantly better in quality, proving that it offers real, practical value over clinical intuition alone. By making four decades of scientific evidence freely accessible and searchable, DEPP aims to empower healthcare professionals worldwide to deliver safer, more effective exercise guidance. We invite clinicians, educators, and researchers to explore the platform and put this resource to use at http://depp.sysbio.org.cn.

## Introduction

Engaging consistently in physical activity (PA) plays a critical role in preventing and slowing the progression of diabetes mellitus (DM). This proactive approach is promising for improving health outcomes, particularly through optimizing glycemic control and reducing the risk of cardiovascular disease (CVD) [1,2]. To translate this evidence into practice, clinical guidelines often rely on the FITT-VP principle—a framework outlining the frequency, intensity, time, type, volume, and progression of exercise [3–10]. However, while effective for general prescription, this framework offers insufficient guidance on safety measures and contraindications, particularly for patients with diabetes-related complications. Moreover, the “one-size-fits-all” approach fails to address individual variability in exercise response and patient preferences, which are critical for long-term adherence [11–13].

The implementation of PA in diabetic populations faces unique and personalized challenges. Different exercise modalities yield distinct effects: walking is associated with lower energy expenditure and hypoglycemia risk, while cycling or running may lead to greater glucose depletion [14,15]. Fear of hypoglycemia—and actual hypoglycemic events during or after exercise—are significant barriers to adherence [16,17]. Furthermore, patients often present with comorbidities such as cardiovascular disease, neuropathy, or retinopathy, which require careful exercise modifications tailored to individual health profiles [18]. Physical inactivity is a risk factor for all-cause and cardiovascular disease deaths [19,20]. Although personalizing exercise prescriptions according to patient preferences, fitness levels, and glucose variability can improve outcomes [21], clinicians lack efficient tools to access and integrate such complex, evidence-based recommendations into their decision-making process.

To address these limitations, we developed the Diabetes Exercise Prescription Protocol (DEPP) knowledge base, a comprehensive knowledge base manually derived from 529 published articles and comprising 766 structured exercise prescriptions. Critically, DEPP extends the conventional FITT‑VP framework by introducing the FITT‑VP‑WC principle, which explicitly integrates Warnings and Contraindications as core safety components. This extension enables clinicians to assess not only what exercise to prescribe, but also for whom it is safe.

**Fig 1:**
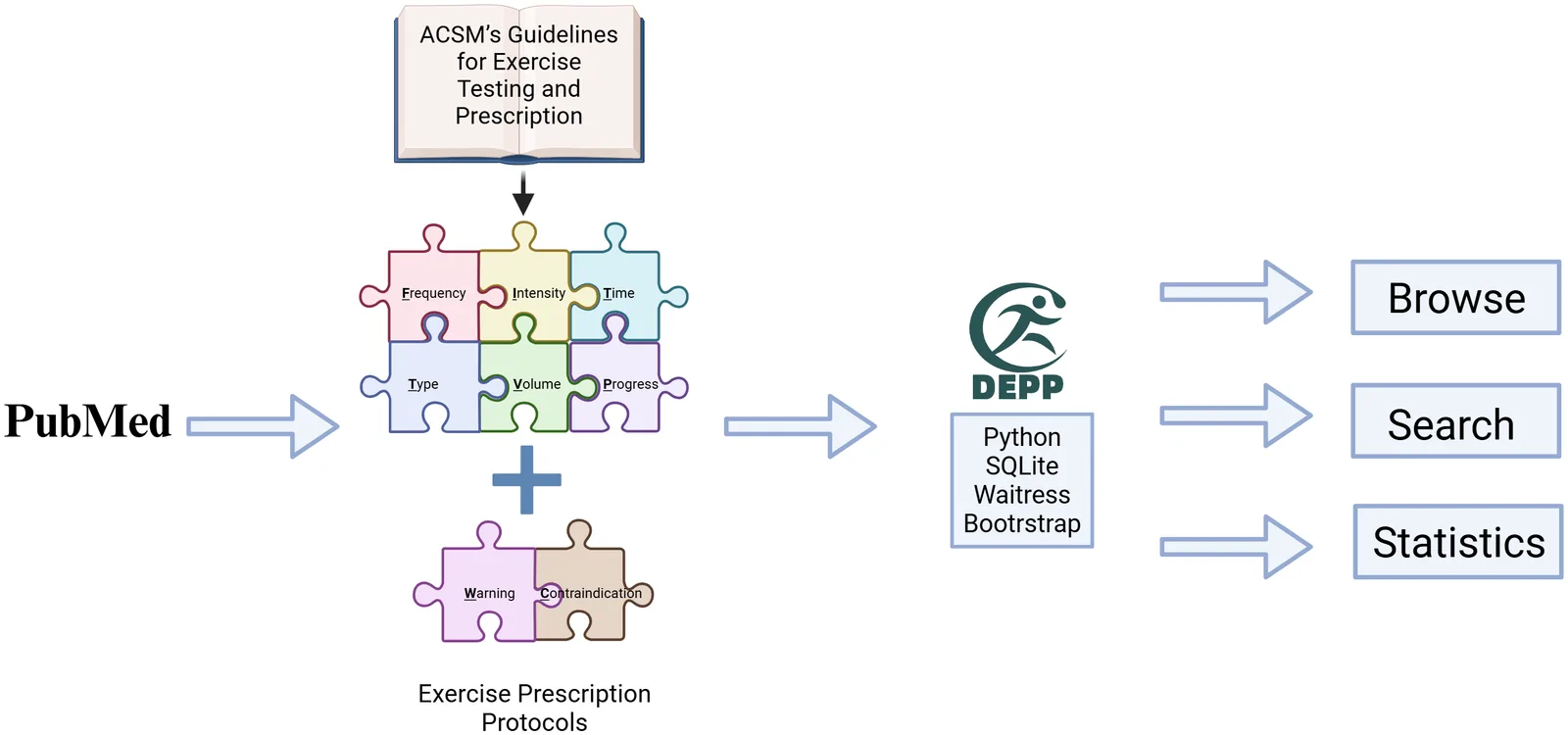
Diabetes Exercise Prescription Protocol (DEPP) knowledge base. DEPP is developed from published literature to structure exercise prescriptions using the FITT-VP-WC principle, integrating Warnings and Contraindications for safety.

DEPP is currently released as Version 1.0, functioning as a reference repository that enables users to query and review existing evidence-based exercise prescriptions based on individual patient conditions, risks, and clinical contexts. Rather than automatically generating a final prescription, DEPP empowers clinicians to make informed selections through side‑by‑side protocol comparison, integrating both evidence strength and safety considerations. While the long‑term vision includes developing automated, patient‑specific recommendation algorithms, the current version deliberately focuses on establishing a solid, transparent evidence foundation, a necessary prerequisite for any future intelligent system. Fig 1 illustrates the data sources and query functions of DEPP.

## Materials and Methods

### Data source and processing

**Fig 2:**
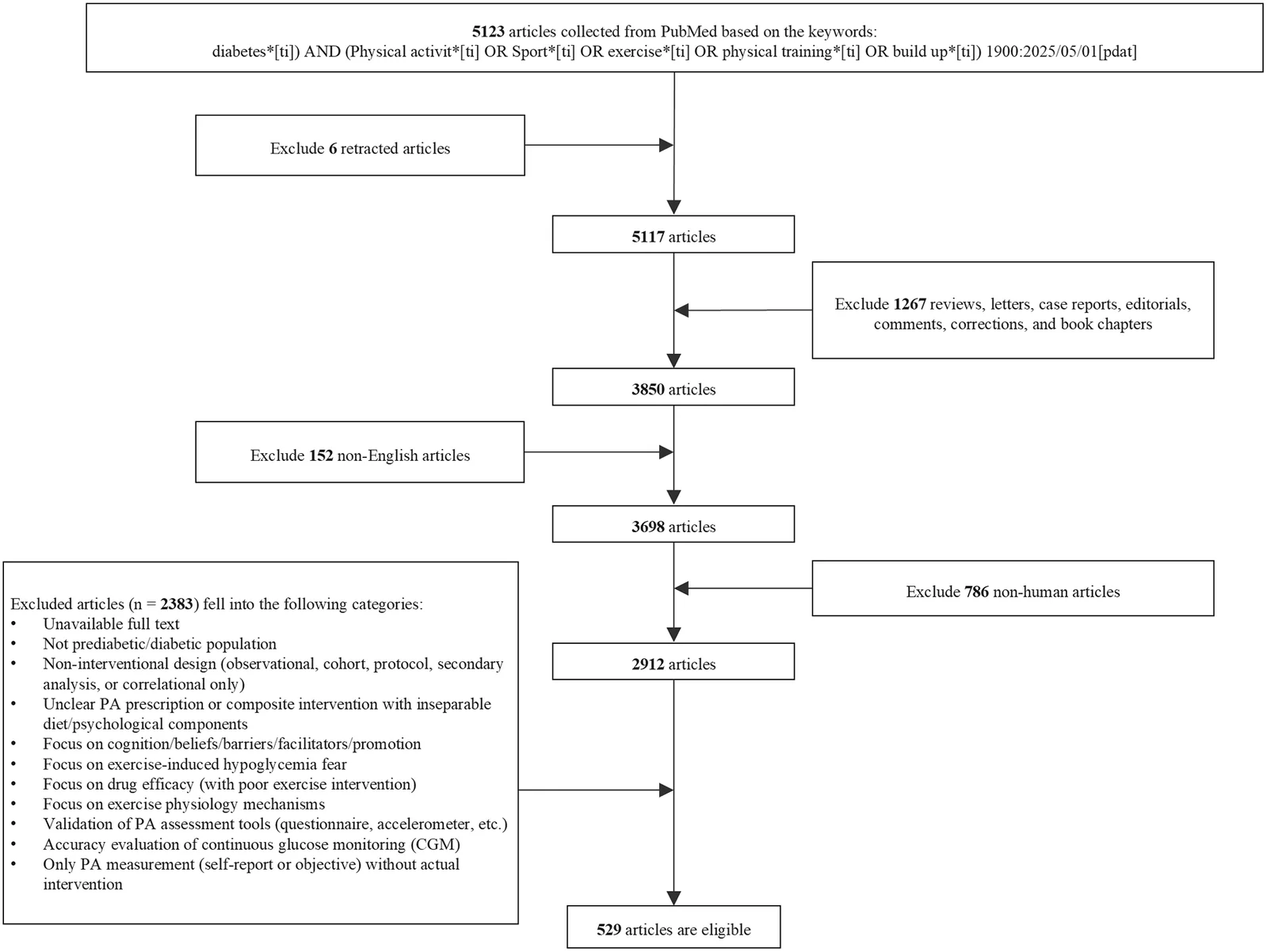
Flowchart of literature screening and data extraction for knowledge base construction.

We performed a targeted literature search of the PubMed database to identify peer‑reviewed studies published in English up to May 1, 2025, as the primary source for knowledge extraction. The search strategy employed in the PubMed database was defined as follows: “(diabetes*[ti]) AND (Physical activit*[ti] OR Sport*[ti] OR exercise*[ti] OR physical training*[ti] OR build up*[ti]) 1900:2025/05/01[pdat]”. We restricted the search to title fields and to PubMed alone to prioritize precision and feasibility for manual curation, aiming to build a version 1.0 knowledge base with high‑certainty, directly relevant records rather than exhaustive coverage. Subsequently, a manual review of 529 articles (published between 1986 and 2025) led to the identification and organization of pertinent knowledge bases. All screening was performed by a single reviewer, prompting a post‑hoc quality check (see next subsection). The flowchart for the collection of studies can be found in Fig 2. All included articles were double‑checked to confirm their compliance with the established inclusion and exclusion criteria.

Based on our team’s Exercise Medicine Ontology [22] and in-depth discussions, we selected information specific to diabetes and exercise prescriptions to develop a comprehensive knowledge base. This knowledge base includes nine tables: Reference information, DM information, Health baseline information, Laboratory testing information, Exercise risk assessment, Physical fitness evaluation, PA intervention protocol, PA effect evaluation, and PA adverse events. These tables cover 111 variables, each accompanied by a detailed definition and standardization protocol. For more information, please refer to the help page on the designated website.

For the exercise prescriptions, we focused on the “FITT-VP-WC” principle: Frequency, intensity, time, type, volume and progression, plus warning and contraindication. For “FITT-VP”, we followed the guideline of ACSM (American College of Sports Medicine) [23]. Warning refers to the precautions to be observed before, during, and after the implementation of an exercise prescription protocol. This includes, but is not limited to, preparatory activities prior to exercise and the configuration of necessary environmental conditions; movements that should be monitored or avoided during exercise execution, such as preventing extreme stretching or joint overextension [24,25]; and post-exercise measures for the prevention of adverse events [26,27]. Contraindications denote the contraindications for exercise, primarily derived from the inclusion and exclusion criteria of the study population. Examples include individuals with severe cardiac conditions or those with absolute contraindications to specific forms of exercise [28–30]. To address the issue of incomplete reporting in the retrieved literature, we supplemented every protocol lacking explicit WC content with standardized, guideline-based generic warnings and contraindications.

In line with classification standards from the ACSM and expert consensus [23], we categorized exercises into five types: aerobic (cardiorespiratory endurance), resistance, flexibility (stretching), neuromotor, and combination exercises (which involve two or more types). We graded the level of evidence for each included study according to the Oxford Centre for Evidence-Based Medicine: Levels of Evidence (March 2009) [31]. We retained this version because its finer sub‑levels for RCT evidence (1a–1c) better suit our study, and the 2011 update does not alter our core conclusions.

S1 Appendix contains both the detailed variable definitions and the entity–relationship diagram. For any variable not extractable due to missing reporting or ambiguous wording, we assigned ‘NA’ to avoid subjective imputation and to preserve fidelity to the original publications.

### Literature screening and post-hoc quality assurance

As noted above, the initial screening was performed by a single reviewer. To assess the reliability of this process, we conducted a post-hoc quality check to assess the reliability of this process. We randomly drew a 10% sample (n = 291) from the 2,912 records that passed the hard eligibility criteria. A second, independent reviewer, who was not involved in the original screening, re-evaluated this sample against the same inclusion/exclusion criteria, blinded to the original decisions. All discrepancies between the original and the second reviewer's decisions were discussed and resolved through consensus, with a third reviewer adjudicating when an agreement could not be reached.

To verify extraction reliability, we randomly selected 10% (n = 53) of the 529 included articles. An independent reviewer, not involved in the original extraction, re‑extracted the FITT‑VP‑WC elements from these articles and compared them with the original data. For articles with multiple protocols, all were compared, but error counting remained at the article level (eight elements per article). An error in any protocol for a given element counted as one error for that element; complete omission of a protocol counted as errors for all eight elements. Discrepancies were resolved by consensus, with a third reviewer adjudicating when needed. This check focused solely on these core prescription elements, as other variables were transcribed directly from the source with minimal interpretation and thus had lower error risk.

### System architecture and informatics implementation

The platform follows a three‑tier B/S architecture built with Flask, SQLite, Waitress, and Bootstrap [32]. To ensure maintainability and scalability, the backend uses Flask’s application factory pattern with six independent blueprints, enabling modular feature addition and multi‑environment configuration. At the data layer, a central SQL view encapsulates JOINs across five core tables, insulating business logic from schema changes. The database schema defines clear entity relationships: each protocol is linked to a source study via PMID, assigned an internal protocol_number, and associated with multi‑valued clinical dimensions to support fine‑grained filtering.

On the Search page, we employ a rule‑based weighted similarity scoring system. Multi-value fields are tokenized and normalized via delimiters. The base score measures the overlap between user-specified filters and each database record, comparable to Jaccard similarity but with weights stratified by clinical significance. This scheme prioritizes critical diagnostic attributes, assigns lower weights to general risk factors, and imposes a fixed penalty for any unmatched requested risk factors. The retrieval layer processes multiple front-end query dimensions by constructing parameterized conditions with bound inputs to ensure secure execution. An adaptive dual-path strategy governs the workflow: an initial row count determines the route. When the number of exact candidates exceeds a set threshold, server-side pagination is triggered. Conversely, if few exact matches are found, the system relaxes constraints, rescans the entire dataset, scores every entry, and merges top similar results alongside exact ones, visually distinguishing them on the interface.

To reduce repeated aggregation load, 21 chart endpoints use process‑level JSON caching (TTL 300 s) with thread safety. Front‑end tables implement server‑side pagination with configurable page sizes, while a three‑tier CDN fallback ensures resource availability. Filter states are encoded in URL query strings for shareable views, and detail modals lazy‑load content via AJAX. All functionality is exposed through more than 40 RESTful JSON endpoints with a unified response format. These endpoints support multi‑condition filtering, semantic search, and literature details, while a built‑in cross‑table ID resolution maps five identifier types to PMID, ensuring platform openness and interoperability. Beyond structured querying, the platform features a dedicated knowledge graph module that models the database as a typed entity-relationship network with six node categories and seven relationship predicates, each annotated with evidence PMID and weight. The graph is exposed through RESTful endpoints for overview statistics, neighbor traversal, and full graph-data export, while individual protocols are serializable as JSON-LD documents for linked-data interoperability.

These implementation choices directly support the FAIR principles [33]. To ensure findability, each extracted exercise protocol is linked to its source study via the PMID, which serves as a standard external identifier, and each protocol is assigned a unique internal Protocol ID for local management, with the structured metadata (diabetes type, intervention setting, key exercise parameters) enabling the multi‑field semantic search described above. For accessibility, data is stored in XLSX format, and the access protocols are explicitly described in the accompanying documentation. Interoperability is achieved at both the system level (through the standardized RESTful JSON APIs) and the data level (by standardizing exercise variables using consistent definitions and measurement units across the entire dataset). Regarding reusability, every data point is linked to its original source and extraction context, and the conditions for data use are clearly stated, allowing other researchers to legitimately reuse the knowledge base for purposes such as meta‑analyses, clinical guideline development, or comparative effectiveness studies.

### Trends in FITT-VP-WC reporting completeness and sensitivity analyses

We present a formal evaluation of temporal trends in reporting completeness using univariate logistic regression, with study year (scaled per 10‑year increment) as the independent variable. Separate models were fitted for three binary outcomes: complete reporting of all eight FITT‑VP‑WC components (8/8), the six FITT‑VP components (6/6), and the four core FITT components (4/4). Results are reported as odds ratios (ORs) with 95% confidence intervals (CIs).

To assess whether temporal trends differed consistently across clinical and intervention characteristics, we performed subgroup interaction analyses using binary logistic regression models that included study year (per 10‑year increment), the grouping variable, and their interaction term (year × group). Separate models were constructed for diabetes type (reference: T2DM) and exercise modality (reference: Aerobic). Global interaction p‑values were obtained via Wald tests. Group‑specific interaction effects are presented as ORs with 95% CIs. Subgroups with fewer than 10 studies were excluded from these analyses.

To examine the robustness of our temporal findings against potential biases in study inclusion and classification, we conducted a series of sensitivity analyses using the same logistic regression framework (per 10‑year increment). These included: [1] restricting the publication window to studies published after 2000, 2010, 2016, and 2021; [2] refining diabetes type definitions by excluding studies with multiple diabetes types or unspecified (NA) types; and [3] stratifying by study design (restricted to RCTs only, non‑RCTs only, and studies with a known design only). Each sensitivity model was fitted separately for the 8/8, 6/6, and 4/4 outcomes.

### Usability evaluation

The usability, loyalty, and satisfaction of the DEPP were assessed using the modified System Usability Scale (SUS) [34,35] and Net Promoter Score (NPS) [36]. The evaluation involved 12 sports physicians from 10 institutions across China (West China Hospital, Sichuan University; West China Lecheng Hospital, Sichuan University; West China Lecheng Hospital, Sichuan University (Jinjiang Campus); Shang Jin Hospital of West China Hospital, Sichuan University; West China Lecheng Hospital, Sichuan University (Wenjiang Campus); West China Emei Hospital, Sichuan University; Wuhou Health Examination Center of West China Hospital; Tian Fu Hospital of West China Hospital, Sichuan University; West China Hospital, Sichuan University; Xizang Hospital of West China Hospital, Sichuan University; and West China Xiamen Hospital of Sichuan University) and 16 postgraduate students from the West China School of Public Health at Sichuan University. The sports physicians were all well versed in exercise prescription, whereas the postgraduate students possessed only limited prior knowledge in this area. Their feedback, therefore, offered valuable novice‑perspective insights into potential usability issues, complementing the expert assessments.

### Vignette‑based comparative validation

To preliminarily assess the clinical utility of DEPP, we conducted a vignette‑based comparative study. Twenty clinicians were recruited from 10 institutions, two from each. These institutions are the same as those described in the Usability Evaluation section. The participants were randomly assigned to two groups (n = 10 each): Group A generated exercise prescriptions with access to the DEPP knowledge base, while Group B produced prescriptions without any decision support tool, relying solely on clinical judgment.

Each clinician independently developed written exercise prescriptions for three standardized simulated patient cases: (1) a middle‑aged obese patient with type 2 diabetes and hypertension; (2) an elderly patient with long‑standing type 2 diabetes, knee osteoarthritis, CKD stage 3, and risk of hypoglycemia; and (3) a young patient with type 1 diabetes using an insulin pump and recurrent post‑exercise hypoglycemia. The case order was randomized to avoid sequence effects.

Prescriptions were scored using a predefined rubric covering four dimensions: Medical Safety & Compliance (40%), Personalization (20%), Actionability (20%), and FITT‑VP Completeness (20%). Each dimension was rated on a 1–5 scale, and a weighted total score was calculated (range 0–25). Scoring was performed in two steps: (1) GPT‑5.4‑mini was prompted to assign dimension scores and provide rationales for each prescription; (2) two research team members independently reviewed all AI‑generated scores, resolved discrepancies by consensus, and adopted the manually verified scores as final.

Comparisons between groups were made using Mann‑Whitney U tests to determine whether DEPP assistance significantly improved prescription quality.

### Ethics statement

The assessment protocol of the DEPP (including SUS, NPS, and the exercise prescription formulation for virtual patients) was reviewed and approved by the Institutional Review Board of West China Hospital, Sichuan University.

## Results

### Screening outcomes and reliability assessment

To evaluate the reliability of the screening process, we performed a post-hoc quality check on a 10% random sample (n = 291) during revision. Against the consensus standard, the original screening decisions showed an overall error rate of 1.37% (4/291, all false negatives). The independent reviewer who performed the quality check also made 3 errors (1 false negative and 2 false positives) relative to the consensus. The inter-reviewer agreement was excellent: observed agreement was 96.22%, and Cohen‘s kappa was 0.92, indicating almost perfect agreement beyond chance.

For the post‑hoc quality check on core prescription elements, we randomly selected 53 articles and re‑extracted the eight FITT‑VP‑WC components by an independent reviewer. Among the 424 total elements, the original extraction contained 29 erroneous entries (6.8%), whereas the re‑extraction contained 27 erroneous entries (6.4%) when compared against the final consensus standard after adjudication.

### Applicable population characteristics

The DEPP knowledge base integrates data from 529 studies, comprising a total of 766 exercise prescription protocols. In terms of sex distribution, female-only participants account for 6.6% of the data, male-only for 4.4%, and studies including both sexes represent the majority (87.9%). By age group, the largest share is adults‑only (48.7%), followed by the combined adults and older adults group (33.8%), and then older adults‑only (6.4%). Regarding body mass index (BMI) categories, overweight and obese individuals constitute the two largest groups, included in 59.0% and 56.9% of the studies, respectively. Among cardiovascular risk factors, age-related risk was reported in 73.7% of the protocols. Other frequently noted risk factors include smoking (14.9%), hypertension (26.3%), sedentary lifestyle (8.9%), and lipid metabolism disorders (37.1%). Type 2 diabetes was the most common condition represented, comprising 73.7% of the study populations. A summary of the demographic characteristics is provided in Table 1.

**Table 1:** Frequency of Population Characteristics Across Studies.

Characteristics	Studies, n (%)
**Total**	529 (100.0)
**Sex**	
Female and male	465 (87.9)
Female-only	35 (6.6)
Male-only	23 (4.4)
NA	6 (1.1)
**Age group**	
Children and adolescents-only	19 (3.6)
Adults-only	258 (48.7)
Older adults-only	34 (6.4)
Adults and older adults	179 (33.8)
Others	39 (7.4)
**BMI group**	
Include healthy weight (18.5–24.99 kg/m2)	155 (29.3)
Include Overweight (25–29.99 kg/m2)	312 (59.0)
Include Obese (≥30 kg/m2)	301 (56.9)
**Cardiovascular risk factors**	
Age (male≥45, female≥55)	390 (73.7)
Smoke (current smoker or the quit smoking time less than 6 months or second smoking)	79 (14.9)
Hypertension (SBP≥140 mmHg or/and DBP≥90 mmHg)	139 (26.3)
Sedentary	47 (8.9)
Lipid metabolism disorder (TC, TG, LDL-C, HDL-C)	196 (37.1)
**Diabetes mellitus type**	
IGT	2 (0.4)
GDM	20 (3.8)
T1DM	75 (14.2)
T2DM	390 (73.7)
Multiple types	26 (4.9)
NA	16 (3.0)
**Diabetes mellitus complication**	37 (7.0)
**Duration of diagnosed years**	
Include newly diagnosed (≤1 years)	100 (18.9)
Include 2 - 9 years	283 (53.5)
Include ≥10 years	239 (45.2)
**Using continuous glucose monitoring devices**	34 (6.4)
**Oxford Evidence Level**	
1a, 1b, 1c	173 (32.7)
2a, 2b, 2c	250 (47.3)
3a, 3b	102 (19.3)
4	4 (0.8)

BMI, body mass index; SBP, systolic blood pressure; DBP, diastolic blood pressure; TC, total cholesterol; TG, triglyceride; LDL-C, low-density lipoprotein cholesterol; HDL-C, high-density lipoprotein cholesterol; IFG, impaired fasting glucose; IGT, impaired glucose tolerance; GDM, gestational diabetes mellitus; T1DM, type 1 diabetes mellitus; T2DM, type 2 diabetes mellitus.

### Characteristics of exercise prescription protocols

**Fig 3:**
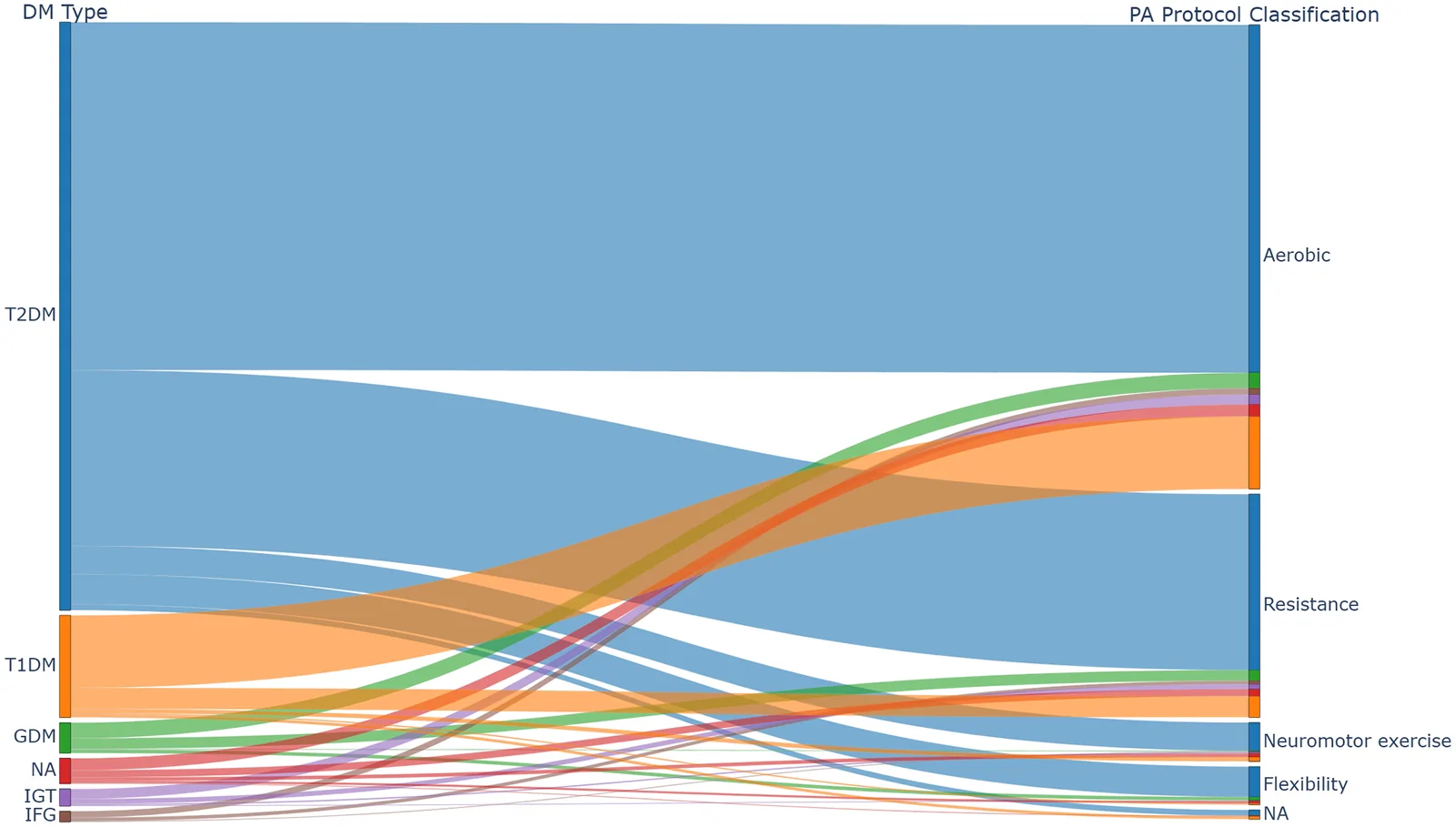
Relationship between diabetes mellitus (DM) type and classification of physical activity (PA) protocols.

A total of 766 exercise prescription protocols were extracted from the included articles, with each protocol comprising 23 distinct elements. Detailed descriptions of these elements are available on the “Help” page of the website. Of these protocols, 23.6% required an exercise risk assessment prior to implementation, 74.7% required medical supervision during implementation, and 49.0% involved exercise intensity monitoring. Aerobic (cardiorespiratory endurance) exercises constituted the most common type of PA (55.6%), followed by combined training (27.3%), resistance exercises (13.7%), neuromotor exercises (1.2%), and flexibility (stretching) exercises (0.5%). Further characteristics of the exercise prescription protocols are summarized in Table 2. Fig 3 shows the relationship between DM type and classification of physical activity protocols in DEPP.

**Table 2:** Characteristics of physical activity protocols.

Characteristics	Protocols, n (%)
**Total**	766 (100.0)
**General descriptive information**	
Need exercise risk assessment ^a^	181 (23.6)
Need medical supervision ^b^	572 (74.7)
Need exercise intensity monitoring ^c^	375 (49.0)
For patients with diabetic complications	45 (5.9)
For patients with 1 cardiovascular risk	272 (35.5)
For patients with 2 cardiovascular risks	174 (22.7)
For patients with 3 cardiovascular risks	85 (11.1)
For patients with 4 cardiovascular risks	42 (5.5)
For patients with 5 cardiovascular risks	40 (5.2)
**Classification**	
Aerobic (cardiorespiratory endurance)	426 (55.6)
Resistance	105 (13.7)
Neuromotor exercise	9 (1.2)
Flexibility (stretching)	4 (0.5)
Combined (any combination of other exercise)	209 (27.3)
NA	13 (1.7)
**Physical activity intensity**	
Include very light	21 (2.7)
Include light	91 (11.9)
Include moderate	404 (52.7)
Include vigorous	299 (39.0)
Include near-maximal to maximal	26 (3.4)

FITT-VP-WC, Frequency, Intensity, Time, Type, Volume, Progress, Warning, Contraindication; IQR, interquartile range

^a^Assigned if the study mentioned pre-exercise screening (e.g., PAR-Q, medical history) or baseline physiological testing (e.g., cardiorespiratory fitness assessment, maximal or submaximal exercise testing) to evaluate risk.

^b^Assigned if the intervention required the presence or involvement of certified clinicians or healthcare professionals.

^c^Assigned if the protocol utilized real-time physiological feedback (e.g., heart rate, RPE, or oxygen consumption) to adjust workload.

#### FITT-VP-WC statistics.

**Fig 4:**
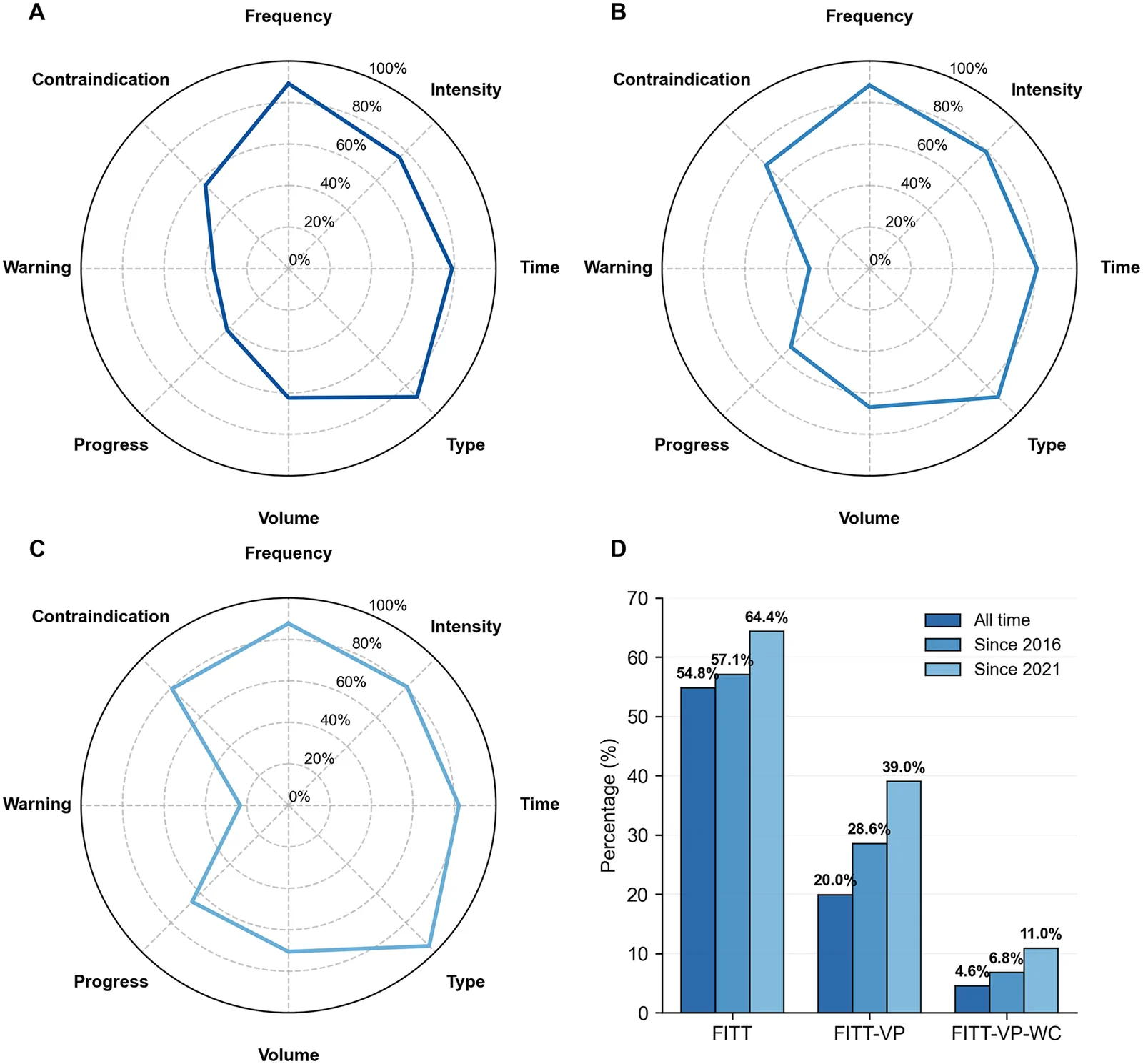
“FITT-VP-WC” principle of protocols. “FITT-VP-WC”: frequency, intensity, time, type, volume, progression, warning and contraindication. **(A)** Coverage of FITT-VP-WC for all time. **(B)** Coverage of FITT-VP-WC since 2016. **(C)** Coverage of FITT-VP-WC since 2021. **(D)** Coverage of fully defined FITT, FITT-VP, and FITT-VP-WC.

We first assessed the reporting completeness of the eight elements of the FITT‑VP‑WC framework across all included protocols. Among these eight elements, frequency was the most reported (89.2% of protocols), while warning had the lowest coverage (36.0%), followed closely by progress (42.0%). When examining the completeness of protocol reporting, 54.8% of protocols fully defined the core FITT components. This proportion was 20.0% for the extended FITT-VP combination and 4.6% for the comprehensive FITT-VP-WC set. As shown in Fig 4A-4C, reporting rates for all elements except frequency and warnings were higher in protocols published since 2021 than in the overall dataset. A corresponding increase was also observed in the proportion of protocols that fully defined FITT, FITT-VP, and FITT-VP-WC (Fig 4D).

Among the included protocols (1986–2024; data from 2025 were incomplete and therefore excluded from the analysis), we observed a significant per‑decade increase in the odds of complete reporting for the full FITT‑VP‑WC framework (8/8: OR = 3.024, 95% CI: 1.552–5.893, P = 0.001) and for the FITT‑VP components (6/6: OR = 2.370, 95% CI: 1.714–3.276, P < 0.001). However, no significant temporal change was detected for the basic FITT components alone (4/4: OR = 1.113, 95% CI: 0.898–1.381, P = 0.329).

The temporal improvement in FITT‑VP (6/6) and FITT (4/4) completeness differed significantly across diabetes types (global interaction P = 0.025 and 0.031, respectively), but not for the full FITT‑VP‑WC (8/8; global P ≈ 1.000). Specifically, for the 6/6 outcome, studies in T1DM showed a significantly slower per‑decade improvement than those in T2DM (interaction OR = 0.378, 95% CI: 0.173–0.828, P = 0.015). For the 4/4 outcome, GDM studies demonstrated a significantly faster improvement over time compared with T2DM studies (interaction OR = 6.377, 95% CI: 1.149–35.383, P = 0.034).

A significant interaction between exercise type and year was observed only for the FITT (4/4) outcome (global P = 0.034). Resistance training studies exhibited a significantly faster per‑decade increase in reporting completeness compared with aerobic studies (interaction OR = 3.091, 95% CI: 1.300–7.351, P = 0.011). No significant interaction was detected for combined training versus aerobic training (P = 0.362), nor for any exercise‑type interaction with the FITT‑VP (6/6) or FITT‑VP‑WC (8/8) outcomes (global P > 0.05 for both).

In sensitivity analyses, the primary temporal trends remained consistent across most scenarios. When restricting the analysis to studies published from 2010 onward, the per‑decade improvement became significant for the basic FITT (4/4) components (OR = 1.809, 95% CI: 1.237–2.645, P = 0.002), alongside significant increases for 8/8 (OR = 4.872, P < 0.001) and 6/6 (OR = 3.338, P < 0.001). Variations in diabetes type classification (excluding multiple or unspecified types) did not materially alter the effect estimates for any outcome. Regarding study design, the temporal improvement in complete 8/8 reporting remained significant in both RCTs (OR = 4.235, 95% CI: 1.695–10.578, P = 0.002) and non‑RCTs (OR = 3.566, 95% CI: 1.031–12.339, P = 0.045). However, the improvement in FITT (4/4) reporting reached significance only in the RCT subset (OR = 1.437, 95% CI: 1.046–1.972, P = 0.025), but not in non‑RCTs (OR = 0.938, 95% CI: 0.680–1.295, P = 0.699).

**Fig 5:**
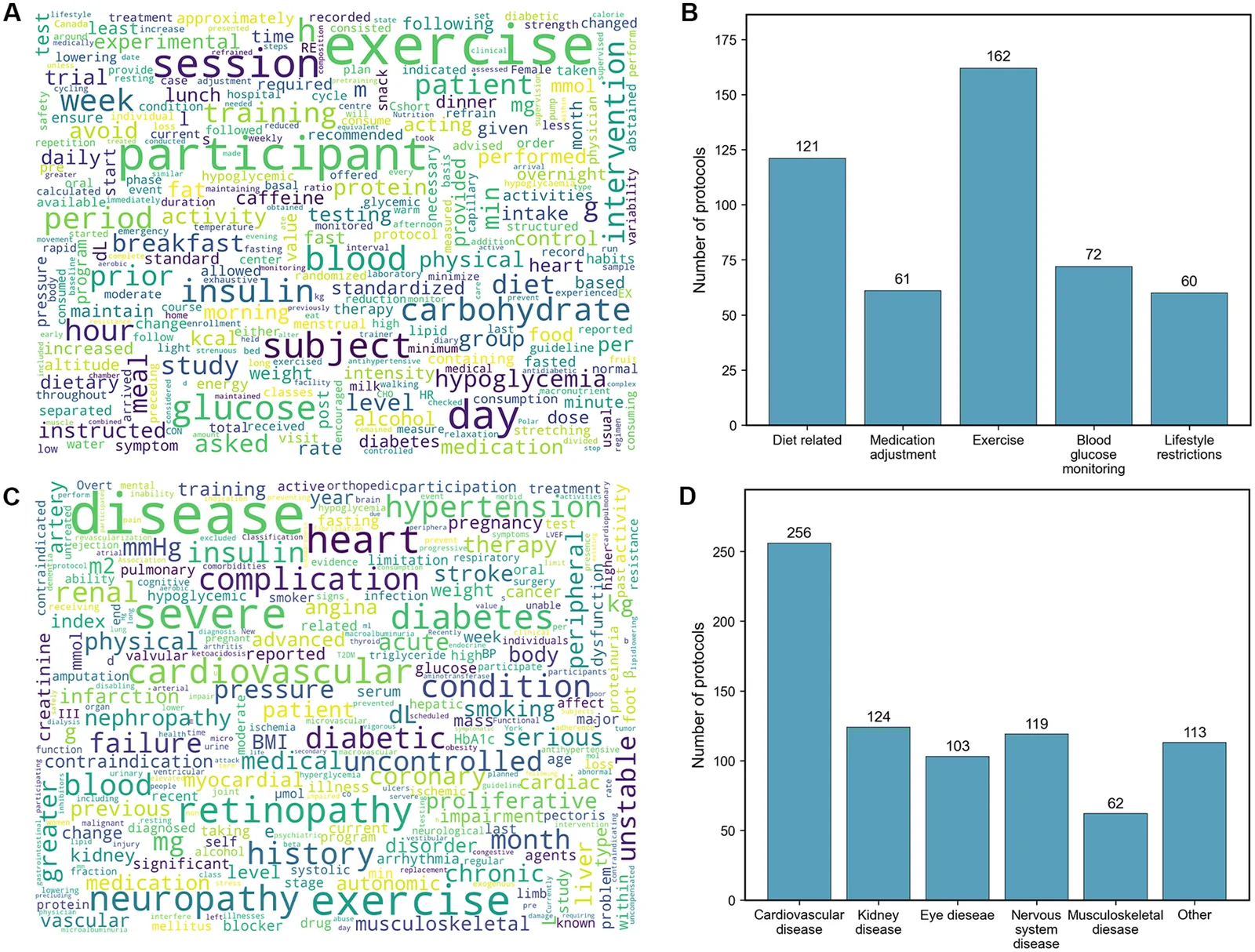
Warning and contraindication in Diabetes Exercise Prescription Protocol (DEPP) knowledge base. (A) Word cloud of warning. (B) Category of warning. (C) Word cloud of contraindication. (D) Category of contraindication.

Warnings identified in the DEPP were categorized into five types: diet-related, medication adjustment, exercise, blood glucose monitoring, and lifestyle restrictions. Some protocols contained two or more of these categories. The corresponding word cloud for warnings is presented in Fig 5A, and the categorical distribution is shown in Fig 5B. Similarly, contraindications were classified into the following groups: cardiovascular disease, kidney disease, eye disease, nervous system disease, musculoskeletal disease, and other conditions. The word cloud for contraindications is provided in Fig 5C, with the breakdown of categories displayed in Fig 5D.

#### Website interface.

The DEPP knowledge base includes five main pages: “Home”, “Browse”, “Search”, “Statistics”, and “Help”. Home page introduces the knowledge base to the users. In Browse page, users can browse DM information, health baseline information, laboratory testing information, exercise risk assessment, physical fitness evaluation, PA intervention protocol, adverse events evaluation and prevention protocol, and PA effect evaluation from the studies and protocols. In search page, users can select information by setting the restrictions of patient’s situation, such as DM type, DM complications, sex, BMI, cardiovascular risks. Statistics illustrate the whole data statistics by studies or protocols. The last page, help, provides additional resources and documentation to help users better understand and utilize our platform.

### Usability evaluation results

The overall SUS score was 80.18 ± 7.93. Sports physicians achieved a higher mean SUS score (83.13) than postgraduate students (77.97), with a mean difference of 5.16. For user loyalty, the overall NPS was 28.6%. Sports physicians had an NPS of 50.0%, considerably higher than the 12.5% observed among postgraduate students, representing a 37.5‑percentage‑point gap. Complete descriptive statistics and group‑wise distributions are available in Table 3. Statistically, the SUS difference was not significant using Welch’s t‑test (*t*(15.9) = 1.63, *p* = 0.122), while the NPS difference was not (Adjusted‑Wald Z = 1.37, p = 0.170).The complete statistical details for both SUS and NPS are provided in S1 Table.

**Table 3:** The SUS score and the NPS result of DEPP.

	All (n=28)	Sports physicians (n=12)	Postgraduate students (n=16)	Statistics	*P* value
SUS score	80.18 ± 7.93	83.13 ± 9.89	77.97 ± 5.42	*t*(15.9) = 1.63^a^	0.122
NPS					
Promoters n (%)	10 (35.7%)	7 (58.3%)	3 (18.8%)		
Passives n (%)	16 (57.1%)	4 (33.3%)	12 (75.0%)		
Detractors n (%)	2 (7.1%)	1 (8.3%)	1 (6.3%)		
NPS score	28.6%	50.0%	12.5%	Z = 1.37^b^	0.170

DEPP, Diabetes Exercise Prescription Protocol knowledge base; SUS, system usability scale; NPS, net promoter score

^a^Welch’s t test; Cohen's d = 0.68.

^b^Adjusted‑Wald test; difference = 37.5 percentage points (95% CI: –12.7 to 71.6).

### Comparative validation outcomes

DEPP significantly improved total prescription scores in all three cases. For Case 1, median scores were 22.0 (DEPP) vs 17.5 (control), U = 96.5, p < 0.001. For Case 2, medians were 21.0 vs 15.0, U = 97.0, p < 0.001. For Case 3, medians were 22.5 vs 14.5, U = 100.0, p < 0.001. All differences were statistically significant, indicating consistent and substantial benefit of DEPP assistance across diverse patient profiles.

## Discussion

### The rationale for expanding FITT-VP to FITT-VP-WC

The established “FITT-VP” principles effectively outline the “what” and “how much” of exercise prescription [37], yet they do not sufficiently address the critical question of “for whom.” It is widely recognized that a standard exercise prescription protocol may not be suitable for every individual. Patients with specific comorbidities or chronic conditions may need to avoid certain types of exercise, while others may require careful modification to prevent overly vigorous activity [4,38]. Recognizing this gap, we argue that warnings and contraindications deserve greater emphasis in exercise prescription, like their established role in clinical pharmacology [39]. Therefore, in developing the DEPP knowledge base, we integrated these safety elements with the conventional “FITT-VP” framework, resulting in the extended “FITT-VP-WC” principles.

Our results underscore the need for this expansion. While core FITT components were fully reported in 54.8% of protocols, the proportion dropped to 20.0% for FITT‑VP and further to 4.6% for FITT‑VP‑WC, indicating that safety elements are rarely documented alongside basic parameters. Encouragingly, temporal analyses showed significant per‑decade increases for complete FITT‑VP (OR = 2.37) and FITT‑VP‑WC (OR = 3.02), while basic FITT showed no overall improvement (OR = 1.11), suggesting that recent gains are driven by better reporting of “VP” and “WC” elements. However, warnings remained the least reported element (36.0%), even lower than progression (42.0%) and contraindications (overall 40.6%). Unlike absolute contraindications, warnings are conditional and context‑dependent, which may make them more easily overlooked; alternatively, some protocols may genuinely not require specific warnings, which partly explains their lower frequency.

Subgroup analyses revealed heterogeneity with important clinical implications. T1DM studies showed slower improvement in FITT‑VP (interaction OR = 0.38) versus T2DM, potentially reflecting the greater complexity and stricter safety precautions required in managing type 1 diabetes, where hypoglycemia risk is more volatile. Conversely, GDM studies demonstrated faster improvement in FITT (interaction OR = 6.38), likely due to the relatively recent establishment of standardized prenatal exercise guidelines that have driven rapid adoption. Resistance training improved faster in FITT reporting than aerobic training (interaction OR = 3.09), which may be attributed to the intrinsically higher complexity of prescribing resistance exercise (e.g., sets, reps, intensity), necessitating more detailed documentation. By study design, the improvement in FITT (4/4) was significant only in RCTs (OR = 1.44), not in non‑RCTs (OR = 0.94), while 8/8 improvement remained significant in both, suggesting that rigorous RCT standards drive core parameter documentation, but comprehensive reporting is gaining ground across all designs.

Collectively, these findings not only validate the necessity of extending FITT‑VP to FITT‑VP‑WC but also inform how this framework should be operationalized in practice. The persistently low warning coverage, despite its upward trend, remains a critical gap that deserves greater emphasis in future guidelines. Conceptually, we operationalize WC as a three-tier gating system: Tier 1 screens for absolute contraindications [40]; Tier 2 applies risk-specific modifications based on patient comorbidities (e.g., hypertension, neuropathy, retinopathy); Tier 3 generates the final, individualized FITT-VP prescription. For instance, a patient with hypertension, peripheral neuropathy, and retinopathy (risks explicitly documented in Lane et al. [41]) might otherwise receive a moderate‑intensity jogging prescription under a conventional approach; with WC evaluation, however, these risks are flagged and the recommendation is safely adjusted to recumbent cycling at a lower intensity. Similarly, a patient with hypoglycemic unawareness (a condition explicitly listed as an exclusion criterion in Kilbride et al. [26]) would receive a standard walking prescription under FITT‑VP alone; WC evaluation, however, flags the heightened risk of unrecognized hypoglycemia during vigorous weight‑bearing exercise, prompting mandatory pre‑ and post‑exercise blood glucose monitoring, availability of rapid‑acting carbohydrates, and a shift to shorter, lower‑intensity sessions or a non‑weight‑bearing modality such as stationary cycling. Fundamentally, this WC framework reorients clinical reasoning from an “effectiveness-first” to a “safety-first” logic. Instead of asking “What exercise is effective?” FITT-VP-WC reframes the question as: “What exercise is effective, but only after safety has been confirmed?” This patient-centered approach ensures that exercise prescription in diabetes care becomes both efficacious and secure.

### Data integrity and usability evaluation

The Diabetes Exercise Prescription Protocol (DEPP) knowledge base is constructed from data extracted from 529 published studies, comprising a total of 766 exercise protocols. These protocols were collected and organized according to the “FITT-VP-WC” principle, which comprises the eight core elements of frequency, intensity, time, type, volume, progression, warning, and contraindication.

The post‑hoc quality checks confirmed the overall reliability of both the screening and data extraction processes. For screening, the original decisions showed an error rate of only 1.37% (4/291) against the consensus standard, with an inter‑reviewer observed agreement of 96.22% and Cohen's kappa of 0.92, indicating almost perfect agreement. For the core FITT‑VP‑WC prescription elements, the original extraction had 29 errors among 424 elements (6.8%), while the independent re‑extraction had 27 errors (6.4%). Notably, our error counting followed a stringent rule: each article contributed eight core elements regardless of the number of exercise protocols it reported; if a protocol was completely omitted, all eight elements for that article were counted as errors. Thus, the error rates we observed represent a conservative upper bound of the true extraction error frequency. All discrepancies identified during the dual‑check were resolved through consensus adjudication, meaning that every detected error was corrected before finalizing the database. These error rates (6.4%–6.8%) are within the range commonly reported in methodological studies of data extraction (0.5% to 15% [42]). To further improve accuracy in future iterations, we plan to implement an LLM‑assisted extraction pipeline with subsequent human verification, which may reduce omission errors and enhance consistency.

The usability evaluation demonstrates that the DEPP knowledge base received favorable initial feedback from its intended users, as reflected by an overall SUS score of 80.18 (within the “excellent” range) and a positive NPS of 28.6%. Notably, sports physicians from 10 different institutions across China yielded a mean SUS of 83.13, while postgraduate students with limited prior knowledge in exercise prescription gave a mean of 77.97; although this difference did not reach statistical significance (p = 0.122), both subgroups rated the system highly. This pattern suggests that the platform's interface and functionality are acceptable to users with varying levels of domain expertise. The non‑significant difference further indicates that the system is not overly specialized, as even novices were able to navigate and use it with relative ease. The inclusion of physicians from multiple institutions enhances the external applicability of these findings, providing preliminary support for the system's usability across diverse clinical settings.

Complementing these usability metrics, the comparative validation across three representative patient cases provided preliminary evidence of the knowledge base’s clinical impact. In all scenarios, ranging from a young type 1 diabetes patient on an insulin pump to an elderly patient with multiple comorbidities, the DEPP‑assisted group achieved significantly higher total prescription scores than the control group (all p < 0.001). This consistent improvement suggests that access to structured, evidence‑based protocols and explicit safety warnings helps clinicians avoid critical omissions, particularly in complex or high‑risk cases. The magnitude of the median differences (ranging from 4.5 to 8.0 points) further indicates a meaningful practical advantage. Together with the favorable SUS and NPS scores, these findings support the DEPP knowledge base as both usable and effective in enhancing the quality and safety of exercise prescriptions.

### Distinctive positioning

The development of the DEPP knowledge base addresses a critical gap in the field of diabetes management, moving beyond conceptual advancement to offer a practical solution for navigating the extensive and fragmented body of evidence on exercise prescription. The sheer volume of literature often leads to information overload [43], posing a significant barrier for clinicians and researchers seeking to implement evidence-based recommendations. DEPP directly mitigates this challenge by consolidating 766 distinct protocols into a single, queryable platform, thereby transforming disconnected findings into actionable and structured knowledge. Unlike high-level consensus guidelines, DEPP functions as a granular, data-driven repository of actual research protocols. This enables users to interrogate the specific exercise interventions that underpin general recommendations, filling a vital niche between principle and practice. For instance, a clinician managing a patient with comorbid hypertension can instantly retrieve and perform a side-by-side comparison of all aerobic protocols tested in this subpopulation, examining critical variations in intensity, session duration, and frequency. This capacity for rapid, targeted querying significantly lowers the barrier to identifying and comparing suitable protocols, thereby facilitating the creation of more personalized and precise exercise prescriptions.

To further contextualize the unique position of DEPP within the broader landscape of digital resources, we compared it with representative existing tools (summarized in Table 4). First, in terms of domain focus, most existing platforms target general populations (Exercise is Medicine [44], ExerciseRx [45], ExRx.net) or cardiovascular diseases (EXPERT [46]), whereas DEPP is exclusively dedicated to exercise prescription for diabetes care. Second, regarding knowledge structure, Exercise is Medicine and ExRx.net provide general guidelines, ExerciseRx relies on physician-prescribed recommendations, exDSS [47] offers session-based digital twin guidance, and EXPERT focuses on cardiovascular protocols. None offers a structured, quarriable repository of FITT-VP parameters derived from primary research, a gap that DEPP directly addresses. Collectively, these distinctions position DEPP as a specialized resource bridging the gap between high-level guidelines and generic fitness tools, offering clinicians a granular, safety-aware knowledge base for personalized exercise prescription in diabetes care.

**Table 4:** Comparison of Exercise Protocol Resources for Diabetes with DEPP.

Tool / Platform	Reference	Domain	Provided protocol	Structured FITT-VP	Safety
Exercise is Medicine	Lobelo et al. (2014)	General	General guidelines	✓	✓
ExerciseRx	Ehde et al. (2025)	General	Exercise prescription prescribed by physician	✓	✓
ExRx.net	ExRx.net	Fitness	General guidelines	✓	✗
exDSS	Young et al. (2024)	Type 1 diabetes	Digital twin‑based pre‑exercise guidance	Single-session	✓
EXPERT	Hansen et al. (2017)	Cardiovascular diseases	Evidence-based protocols	✓	✓
DEPP	This study	Diabetes	Evidence-based protocols	✓	✓

Furthermore, the process of structuring the extracted data using the FITT-VP-WC framework actively promotes standardization within exercise reporting. By quantifying the reporting completeness across these essential elements, DEPP serves as a reflective tool for the research community, clearly identifying areas where documentation must be improved. A notable example is the consistently low reporting of safety warnings, which is critical to ensuring patient safety and enhancing the transparency and replicability of future research.

### Limitations and future directions

This study has several limitations that should be acknowledged. First, our literature search was restricted to English-language articles indexed in PubMed and further limited to title-only screening. This dual restriction, while deliberately chosen for this proof-of-concept version to ensure high precision and consistent quality control, introduces two interrelated biases. The language restriction may under-represent evidence from high-burden regions (China, India, Southeast Asia, and sub-Saharan Africa) where substantial clinical exercise research is published in non-English journals; the title-only restriction may miss relevant studies whose core exercise protocols are not explicitly reflected in their titles. Together, these may limit the representativeness of the protocols included in DEPP, particularly for populations outside Western settings. In future updates, we plan to address these limitations by: (a) expanding to other databases (e.g., Embase, CINAHL, SPORTDiscus); (b) including major regional databases (e.g., CNKI, Wanfang) and non-English literature through native-language collaborators; and (c) adopting broader search strategies covering abstracts and full texts.

Second, the current dimensionality of the DEPP knowledge base remains limited. It does not fully capture the critical interplay between exercise prescriptions and other components of diabetes management, such as pharmacologic interventions, dietary adjustments, and broader lifestyle modifications—a synergy that is essential for optimal patient outcomes [4]. Additionally, while DEPP successfully provides multiple evidence-based prescription options alongside relevant warnings and contraindications, it currently operates as a descriptive knowledge repository rather than a prescriptive decision-support tool. It lacks granular personalization algorithms capable of dynamically integrating individual patient preferences, contextual constraints, and real-time physiological data. The scope of adverse events and preventive actions included is also currently limited. These gaps may limit its practical utility for managing complex patients and will be addressed in future iterations as we transition the knowledge base toward an adaptive clinical decision aid.

Third, several methodological limitations should be noted regarding our evaluation and quality control procedures. We acknowledge that the inter-annotator reliability analysis was conducted retrospectively as a post-hoc quality check rather than being prospectively designed. Although the observed agreement was high, this post-hoc nature limits the ability to fully assess the consistency of the initial annotation process. In future updates, we plan to implement a formal multi-round independent annotation protocol with pre-specified reliability thresholds. Additionally, the usability assessment involved a relatively small sample (n = 28), limiting statistical power, and the non-significant group differences should be interpreted with caution. Although the physicians were recruited from multiple institutions, they were all from affiliated institutions, and the student evaluators came from a single university, which may have introduced bias. Test-retest reliability was not assessed. Future evaluations should include larger and more diverse cohorts to confirm usability.

Finally, DEPP version 1.0 was intentionally designed for healthcare professionals. Consequently, direct patient involvement in its design and evaluation was beyond the scope of this phase. Looking forward, we plan to enhance the platform's search functionality by incorporating similarity-based retrieval algorithms to yield more comprehensive results. More significantly, we intend to deploy large language models trained on the structured protocols within DEPP to assist in generating and personalizing exercise prescriptions, thereby transitioning the knowledge base from a static resource toward an adaptive clinical decision aid [48,49].

## Conclusions

As the first knowledge base with manually annotated exercise prescription protocols, DEPP represents a significant advancement in diabetes care by enabling evidence-based, interpretable, and highly personalized retrieval and comparison of exercise prescription. Its structured framework, based on the extended “FITT-VP-WC” principles, incorporates crucial safety elements (Warnings and Contraindications) alongside established prescription parameters. Building on this foundation, our future work will deploy large language models to intelligently recommend optimal exercise plans. This next step aims to dynamically balance individual clinical characteristics with user preferences, thereby translating the structured knowledge within DEPP into actionable and personalized recommendations.

## Supporting information

S1 Appendix Data extraction criteria and knowledge base design. (PDF)

S1 Table SUS and NPS metrics of DEPP. (XLSX)
