# Supplementary material for: DEPP: A diabetes exercise prescription protocol knowledge base
Source: PLOS Digit Health. 2026 Sep 16;5(9):e0001728. doi: 10.1371/journal.pdig.0001728 (PMC13581003; doi:10.1371/journal.pdig.0001728)
Supplement: Supplementary file 1 [file pdig.0001728.s001.pdf]

Data extraction criteria and knowledge base design

Table Number	Table Name	**Database Name**	Display Name	Meaning	Standardized Extraction Criteria
1	reference	row_id	ID	Manually generated number of observations codes.	NA
1	reference	pmid	PMID	PubMed unique identifier (PMID) assigned to each article as it is added to PubMed (will not meet the NIH guidelines).	NA
1	reference	year	Year	Year of the article was published.	NA
1	reference	title	Title	Title of the article.	NA
1	reference	author	Author	List all authors of the article.	NA
1	reference	journal	Journal	The name of the publication journal.	NA
1	reference	DOI	DOI	Digital object identifier(DOI),a digital object identifier is a tool for identifying digital information, including Internet information.	NA
1	reference	article_type	Article Type	The article type of PubMed.	NA
1	reference	study_design	Study Design	The type of statistical design used in the study.	NA
1	reference	study_time	Study Time	The study intervention time or study period.	NA

1	reference	sample_size	Sample Size	The sample size of the final included research analysis data set.	NA
1	reference	country	Country	The country in which the study population in the article is located.	Internationally recognized ISO 3166-1 code table for countries and regions, see https://www.iso.org/iso-3166-country-codes.html.
1	reference	race_ethnicity	Race/Ethnic	The race/ethnic of study population.	Table B
1	reference	entry_criteria	Entry Criteria	NA	NA
1	reference	exclusion_criteria	Exclusion Criteria	NA	NA
2	protocol_general	pmid	PMID	PubMed unique identifier (PMID) assigned to each article.	NA
2	protocol_general	evidence_level	Evidence Level	Standardized quality grading for the level of evidence in articles, refer to Oxford Centre for Evidence-Based Medicine: Levels of Evidence (March 2009).	Table A
2	protocol_general	strength_of_recommendation	Evidence Level (Stars)	Strength of recommendation visually express for users, for details, please refer to the attached supplementary material Table A.	Table A
2	protocol_general	exercise_risk_evaluation	Exercise Risk Evaluation	Whether exercise risk assessment is required before exercise intervention.	YES;NO

3	dm	—	ID	Manually generated number of observations codes.	NA
3	dm	pmid	PMID	PubMed unique identifier (PMID) assigned to each article.	NA
3	dm	dm_type	DM Type	Classification of diabetes.	Table C
3	dm	duration_of_diagnosed	Duration of Diagnosed	Years of diagnosed diabetes.	NA
3	dm	—	Duration of Diagnosed Std.	Group of diagnosed years.	Newly diagnosed (≤1 year);2~9 years;≥10 years
3	dm	treatment_protocol	Treatment Protocol	Current treatment protocol for population with diabetes.	drug therapy;exercise therapy;diet therapy;health education
3	dm	drug_type_1	Drug Type I	The type of drugs selected by diabetic patients for drug treatment, mainly divided into two categories: oral antidiabetic agents (OADs) and inject hypoglycemic drugs (IHDs).	Table D
3	dm	drug_type_2	Drug Type II	The name of the hypoglycemic drug for diabetic patients.	Table D
3	dm	diabetes_technology	Diabetes Technology	Diabetes technology is the term used to describe the hardware, devices, and software that people with diabetes use to help manage their condition, from lifestyle to blood glucose levels[].	Table E
3	dm	complication	Complication	Accompanying diabetes-related complications.	Table F

3	dm	complication_standardization	Complication Std.	Whether the subject with DM complication.	YES;NO
3	dm	pregnancy	Pregnancy	Whether the subject is pregnant.	YES;NO
4	baseline	id	ID	Manually generated number of observations codes.	NA
4	baseline	pmid	PMID	PubMed unique identifier (PMID) assigned to each article.	NA
4	baseline	base_class_1	Base Class I	The classification of the baseline information of the eligible population.	Table G-I
4	baseline	base_class_2	Base Class II	The secondary classification index of the baseline information of the eligible population.	Table G-I
4	baseline	base_class_3	Base Class III	The tertiary classification index of the baseline information of the eligible population.	Table G-I
4	baseline	variable_type	Variable Type	The classification of variable types, which are mainly divided into three categories.	Numerical; Categorical; Character
4	baseline	classification_of_categorical	Classification of Categorical	The grouping criteria for baseline categorical variables in the original literature.	NA
4	baseline	statistical_indicators	Statistical Indicators	The statistical descriptive indicators of the baseline variables in the original	Mean±S;Median(IQR);n(%);Range

				literature.	
4	baseline	value	Value	The value of the variable (extract the original data of the literature).	NA
4	baseline	unit	Unit	The unit of the variable.	NA
4	baseline	method	Method	The measurement and testing methods of variables (mainly involving variables assessed through scales and questionnaires).	NA
5	baseline2	id	ID	Manually generated number of observations codes.	NA
5	baseline2	pmid	PMID	PubMed unique identifier (PMID) assigned to each article.	NA
5	baseline2	sex	Sex	Sex of the study population.	Male;Female;Both
5	baseline2	age	Age	Age description of the study population as reported in the original literature.	NA
5	baseline2	age_average	Age Average	Average age of the study population.	NA
5	baseline2	age_sd	Age SD	Standard deviation of the age of the study population.	NA
5	baseline2	age_group	Age Group	Standardized age group classification.	Children;Adolescent;Young Adult;Middle-aged;Elderly
5	baseline2	bmi_group	BMI Group	Standardized BMI group classification.	Underweight;Healthy weight;Overweight;Obese

5	baseline2	age_risk	Age Risk	Whether age is a cardiovascular risk factor.	YES;NO
5	baseline2	lipid_metabolism_disorder	Lipid Metabolism Disorder	Whether the subject has lipid metabolism disorder.	YES;NO
5	baseline2	smoking	Smoking	Whether the subject smokes.	YES;NO
5	baseline2	sedentary	Sedentary	Whether the subject has a sedentary lifestyle.	YES;NO
5	baseline2	hypertension	Hypertension	Whether the subject has hypertension.	YES;NO
6	risk	ex_id	ID	Manually generated number of observations codes.	NA
6	risk	pmid	PMID	PubMed unique identifier (PMID) assigned to each article.	NA
6	risk	exercise_risk_evaluation	Exercise Risk Evaluation	Whether to conduct exercise risk assessment before exercise intervention.	YES;NO
6	risk	method	Method	The method of exercise risk evaluation.	Table J
7	detail	protocol_number	ID	Manually generated number of observations codes.	NA
7	detail	pmid	PMID	PubMed unique identifier (PMID) assigned to each article.	NA
7	detail	protocol_number	Protocol Number	Manually generated number of observations codes.	NA
7	detail	protocol_id	Protocol ID	PA intervention protocol identification ID.	NA

7	detail	classification	Classification	Classification of exercise.	Table K
7	detail	protocol_name	Protocol Name	The special name of PA protocol.	Table L
7	detail	frequency_times_days_week	Frequency	Frequency, which refers to how often you exercise (i.e., the number of days/times per week dedicated to an exercise program).	Table M
7	detail	sets_n	Sets (n)	Number of sets of resistance training exercise.	Table M
7	detail	repetition_per_set	Repetition per Set (n)	Number of repetitions per set of resistance training exercise.	Table M
7	detail	rest_between_sets	Rest between Sets (s)	Rest intervals between sets of resistance training exercise.	Table M
7	detail	weight_rm	Weight (RM)	The magnitude of loading (intensity) during resistance training exercise, intensity is most often prescribed as a percentage of an individual's one repetition maximum (1-RM) for a given exercise.	Table M
7	detail	intensity_calculation_indicator	Intensity Calc. Indicator	The calculation index of exercise intensity.	Table N; Table O
7	detail	training_intensity	Training Intensity	The intensity of exercise after the formal start of exercise, consistent with the intensity in	Table M

				the prescribing principles of exercise.	
7	detail	training_intensity_grade	Intensity	The classification of exercise intensity grade.	Table M
7	detail	warm_up_intensity	Warm-up Intensity	The intensity of exercise during warm-up.	Table M
7	detail	recovery_intensity	Recovery Intensity	The intensity of exercise during recovery.	Table M
7	detail	time_min	Time	How long you exercise per time (min).	Table M
7	detail	type	Type	The mode or what kind you exercise.	Table M
7	detail	volume_week	Volume	The total amount of exercise per week or a period time.	Table M
7	detail	progress	Progress	Refers to increasing any of the components of the exercise principle of personalized exercise prescription as tolerated by the individual.	Table M
7	detail	warm_up_stretching_cool_down	Warm-up / Stretching / Cool Down	This part is also an integral part of the exercise prescription, a transitional phase allowing the body to adjust to changing physiologic, biomechanical, and bioenergetic demands.	Table M
7	detail	intertrial_interval	Intertrial Interval	The interval time of interval	Table M

				training protocols.	
7	detail	duration	Duration	The duration of the training protocol.	Table M
7	detail	medical_supervision	Medical Supervision	The implementation of the training protocol requires medical supervision by appropriately trained individuals.	Table M
7	detail	intensity_monitor_method	Intensity Monitor Method	The means or measures taken in the process of implementing the exercise program to monitor the intensity of exercise.	Table M
7	detail	warning	Warning	Special precautions before, during and after the implementation of the exercise protocol.	Table M
7	detail	contraindication	Contraindication	Special groups of people who are not suitable for this exercise protocol.	Table M
7	detail	protocol	Protocol Description	The description of exercise intervention regimens in the original literature.	NA
8	Warning	id	ID	Manually generated number of observations codes, linked to protocol_number in detail table.	NA
8	Warning	diet_related	Diet Related	Whether the warning involves	0 (No);1 (Yes)

				diet-related precautions.	
8	Warning	medication_adjustment	Medication Adjustment	Whether the warning involves medication adjustment.	0 (No);1 (Yes)
8	Warning	exercise	Exercise	Whether the warning involves exercise-related precautions.	0 (No);1 (Yes)
8	Warning	blood_glucose_monitoring	Blood Glucose Monitoring	Whether the warning involves blood glucose monitoring.	0 (No);1 (Yes)
8	Warning	lifestyle_restrictions	Lifestyle Restrictions	Whether the warning involves lifestyle restrictions.	0 (No);1 (Yes)
9	Contraindication	id	ID	Manually generated number of observations codes, linked to protocol_number in detail table.	NA
9	Contraindication	cardiovascular_disease	Cardiovascular Disease	Whether contraindicated for patients with cardiovascular disease.	0 (No);1 (Yes)
9	Contraindication	kidney_disease	Kidney Disease	Whether contraindicated for patients with kidney disease.	0 (No);1 (Yes)
9	Contraindication	eye_disease	Eye Disease	Whether contraindicated for patients with eye disease.	0 (No);1 (Yes)
9	Contraindication	nervous_system_disease	Nervous System Disease	Whether contraindicated for patients with nervous system disease.	0 (No);1 (Yes)
9	Contraindication	musculoskeletal_disease	Musculoskeletal Disease	Whether contraindicated for patients with musculoskeletal disease.	0 (No);1 (Yes)
9	Contraindication	other	Other	Whether other contraindications	0 (No);1 (Yes)

				exist.	
10	effect	ef_id	ID	Manually generated number of observations codes.	NA
10	effect	pmid	PMID	PubMed unique identifier (PMID) assigned to each article.	NA
10	effect	outcome_class_1	Outcome Class I	The primary classification of the PA effect evaluation.	Table O
10	effect	outcome_class_2	Outcome Class II	The secondary classification of the PA effect evaluation.	NA
10	effect	outcome_class_3	Outcome Class III	The third classification of the PA effect evaluation.	NA
10	effect	outcome_class_4	Outcome Class IV	The four-level classification of the PA effect evaluation.	NA
10	effect	statistics_method	Statistics Method	Refers to the statistics of the PA effect evaluation.	NA
10	effect	value	Value	The value of the statistics.	NA
10	effect	control	Control	The control group of the evaluation of PA effect.	NA
10	effect	results	Results	The results of the PA effect evaluation.	NA
10	effect	conclusion	Conclusion	The conclusion of the PA effect evaluation.	NA
11	adverse	ad_id	ID	Manually generated number of observations codes.	NA
11	adverse	pmid	PMID	PubMed unique identifier (PMID) assigned to each article.	NA

11	adverse	adverse_events_classification	Adverse Events Classification	In the evaluation of an exercise intervention, reporting of exercise-induced injuries or any other adverse events. We categorized adverse events by injury type.	Table P
11	adverse	adverse_events	Adverse Events	The name of adverse events.	NA
11	adverse	adverse_event_definition	Adverse Event Definition	Details of definition of adverse events in the paper.	NA
11	adverse	prevention_protocol	Prevention Protocol	Prevention protocol of adverse events in the paper.	NA

The Display name represents the user-facing label shown on the web front-end, which is distinct from the underlying physical column name in the database. Additionally, certain fields are marked for internal system use only and are therefore not rendered on the user interface.

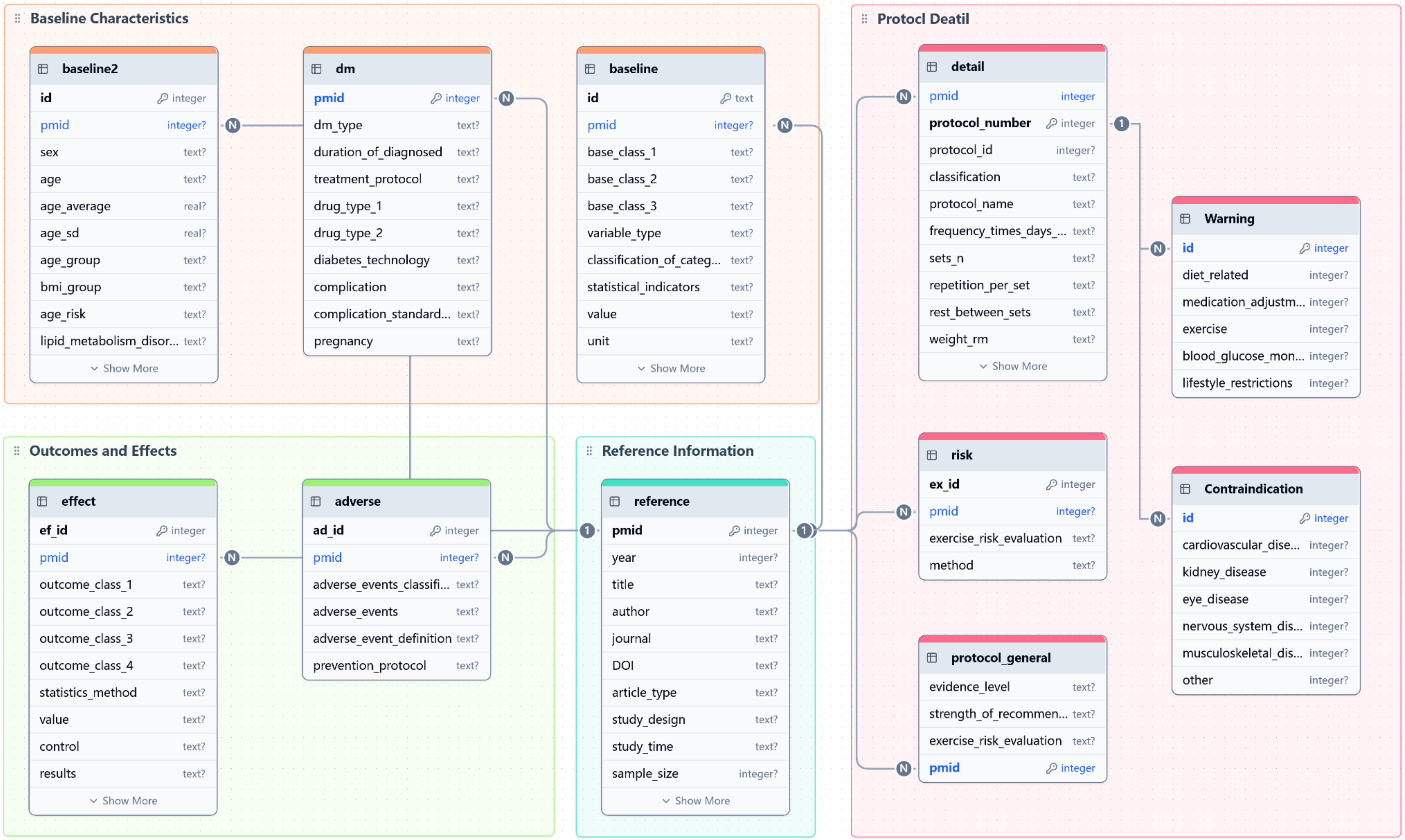
Baseline Characteristics
baseline2
id integer
pmid integer?
sex text?
age text?
age_average real?
age_sd real?
age_group text?
bmi_group text?
age_risk text?
lipid_metabolism_disor... text?
Show More
dm
pmid integer
dm_type text?
duration_of_diagnosed text?
treatment_protocol text?
drug_type_1 text?
drug_type_2 text?
diabetes_technology text?
complication text?
complication_standard... text?
pregnancy text?
baseline
id text
pmid integer?
base_class_1 text?
base_class_2 text?
base_class_3 text?
variable_type text?
classification_of_categ... text?
statistical_indicators text?
value text?
unit text?
Show More
Protocl Deatil
detail
pmid integer
protocol_number integer
protocol_id integer?
classification text?
protocol_name text?
frequency_times_days_... text?
sets_n text?
repetition_per_set text?
rest_between_sets text?
weight_rm text?
Show More
Warning
id integer
diet_related integer?
medication_adjustm... integer?
exercise integer?
blood_glucose_mon... integer?
lifestyle_restrictions integer?
Outcomes and Effects
effect
ef_id integer
pmid integer?
outcome_class_1 text?
outcome_class_2 text?
outcome_class_3 text?
outcome_class_4 text?
statistics_method text?
value text?
control text?
results text?
Show More
adverse
ad_id integer
pmid integer?
adverse_events_classifi... text?
adverse_events text?
adverse_event_definition text?
prevention_protocol text?
Reference Information
reference
pmid integer
year integer?
title text?
author text?
journal text?
DOI text?
article_type text?
study_design text?
study_time text?
sample_size integer?
Show More
risk
ex_id integer
pmid integer?
exercise_risk_evaluation text?
method text?
Contraindication
id integer
cardiovascular_dise... integer?
kidney_disease integer?
eye_disease integer?
nervous_system_dis... integer?
musculoskeletal_dis... integer?
other integer?
protocol_general
evidence_level text?
strength_of_recommen... text?
exercise_risk_evaluation text?
pmid integer
N
1

Table A Classification of level of evidence and strength of recommendation

Classification of level	Strength of recommendation[a]	Therapy / Prevention, Aetiology / Harm	Prognosis	Diagnosis	Differential diagnosis / symptom prevalence study	Economic and decision analyses
1a	★★★★★	SR (with homogeneity[b]) of RCTs	SR (with homogeneity[b]) of inception cohort studies; CDR[c] validated in different populations	SR (with homogeneity[b]) of Level 1 diagnostic studies; CDR[c] with 1b studies from different clinical centres	SR (with homogeneity[b]) of prospective cohort studies	SR (with homogeneity[b]) of Level 1 economic studies
1b	★★★★★	Individual RCT (with narrow Confidence Interval[d])	Individual inception cohort study with > 80% follow-up; CDR[c] validated in a single population	Validating[m] cohort study with good[j] reference standards; or CDR[c] tested within one clinical centre	Prospective cohort study with good follow-up[n]	Analysis based on clinically sensible costs or alternatives; systematic review(s) of the evidence; and including multi-way sensitivity analyses
1c	★★★★★	All or none[e]	All or none case-series	Absolute SpPins and SnNouts[h]	All or none case-series	Absolute better-value or worse-value analyses[k]
2a	★★★★	SR (with homogeneity[b]) of cohort studies	SR (with homogeneity[b]) of either retrospective cohort studies or untreated control groups in RCTs	SR (with homogeneity[b]) of Level >2 diagnostic studies	SR (with homogeneity[b]) of 2b and better studies	SR (with homogeneity[b]) of Level >2 economic studies

2b	★★★★	Individual cohort study (including low quality RCT; e.g., <80% follow-up)	Retrospective cohort study or follow-up of untreated control patients in an RCT; Derivation of CDR[c] or validated on split-sample[g] only	Exploratory[m] cohort study with good[j] reference standards; CDR[c] ” after derivation, or validated only on split-sample[g] or databases	Retrospective cohort study, or poor follow-up	Analysis based on clinically sensible costs or alternatives; limited review(s) of the evidence, or single studies; and including multi-way sensitivity analyses
2c	★★★★	“Outcomes” Research; Ecological studies	“Outcomes” Research		Ecological studies	Audit or outcomes research
3a	★★★	SR (with homogeneity[b]) of case-control studies	/	SR (with homogeneity[b]) of 3b and better studies	SR (with homogeneity[b]) of 3b and better studies	SR (with homogeneity[b]) of 3b and better studies
3b	★★★	Individual Case-Control Study	/	Non-consecutive study; or without consistently applied reference standards	Non-consecutive cohort study, or very limited population	Analysis based on limited alternatives or costs, poor quality estimates of data, but including sensitivity analyses incorporating clinically sensible variations.
4	★★	Case-series (and poor quality cohort and case-control studies[f])	Case-series (and poor quality prognostic cohort studies[l])	Case-control study, poor or non-independent reference standard	Case-series or superseded reference standards	Analysis with no sensitivity analysis
5	★	Expert opinion without explicit critical appraisal, or based on physiology, bench research or “first principles”	Expert opinion without explicit critical appraisal, or based on physiology, bench research or “first principles”	Expert opinion without explicit critical appraisal, or based on physiology, bench research or “first principles”	Expert opinion without explicit critical appraisal, or based on physiology, bench research or “first principles”	Expert opinion without explicit critical appraisal, or based on economic theory or “first principles”

Produced by Bob Phillips, Chris Ball, Dave Sackett, Doug Badenoch, Sharon Straus, Brian Haynes, Martin Dawes since November 1998. Updated by Jeremy Howick March 2009.

NOTES

Users can add a minus-sign “-” to denote the level of that fails to provide a conclusive answer because: EITHER a single result with a wide Confidence Interval OR a Systematic Review with troublesome heterogeneity.

a Strength of recommendation (presented in this article) visually express for users by evidence level presented in this article. Such evidence is inconclusive, and therefore can only generate lower Strength of recommendation.

b By homogeneity we mean a systematic review that is free of worrisome variations (heterogeneity) in the directions and degrees of results between individual studies. Not all systematic reviews with statistically significant heterogeneity need be worrisome, and not all worrisome heterogeneity need be statistically significant. As noted above, studies displaying worrisome heterogeneity should be tagged with a “-” at the end of their designated level.

c Clinical Decision Rule. (These are algorithms or scoring systems that lead to a prognostic estimation or a diagnostic category.)

d See note above for advice on how to understand, rate and use trials or other studies with wide confidence intervals.

e Met when all patients died before the Rx became available, but some now survive on it; or when some patients died before the Rx became available, but none now die on it.

f By poor quality cohort study we mean one that failed to clearly define comparison groups and/or failed to measure exposures and outcomes in the same (preferably blinded), objective way in both exposed and non-exposed individuals and/or failed to identify or appropriately control known confounders and/or failed to carry out a sufficiently long and complete follow-up of patients. By poor quality case-control study we mean one that failed to clearly define comparison groups and/or failed to measure exposures and outcomes in the same (preferably blinded), objective way in both cases and controls and/or failed to identify or appropriately control known confounders.

g Split-sample validation is achieved by collecting all the information in a single tranche, then artificially dividing this into “derivation” and “validation” samples.

h An “Absolute SpPin” is a diagnostic finding whose Specificity is so high that a Positive result rules-in the diagnosis. An “Absolute SnNout” is a diagnostic finding whose Sensitivity is so high that a Negative result rules-out the diagnosis.

i Good, better, bad and worse refer to the comparisons between treatments in terms of their clinical risks and benefits.

j Good reference standards are independent of the test, and applied blindly or objectively to applied to all patients. Poor reference standards are haphazardly applied, but still independent of the test. Use of a non-independent reference standard (where the ‘test’ is included in the ‘reference’, or where the ‘testing’ affects the ‘reference’) implies a level 4 study.

k Better-value treatments are clearly as good but cheaper, or better at the same or reduced cost. Worse-value treatments are as good and more expensive, or worse and the equally or more expensive.

m Validating studies test the quality of a specific diagnostic test, based on prior evidence. An exploratory study collects information and trawls the data (e.g. using a regression analysis) to find which factors are ‘significant’.

l By poor quality prognostic cohort study we mean one in which sampling was biased in favour of patients who already had the target outcome, or the measurement of outcomes was accomplished in <80% of study patients, or outcomes were determined in an unblinded, non-objective way, or there was no correction for confounding factors.

n Good follow-up in a differential diagnosis study is >80%, with adequate time for alternative diagnoses to emerge (for example 1-6 months acute, 1 – 5 years chronic).

Table B The definitions of each racial and ethnic category

Racial and Ethnic Categories	Definitions
American Indian or Alaska Native	A person having origins in any of the original peoples of North and South America (including Central America), and who maintains tribal affiliation or community attachment.
Asian	A person having origins in any of the original peoples of the Far East, Southeast Asia, or the Indian subcontinent including, for example, Cambodia, China, India, Japan, Korea, Malaysia, Pakistan, the Philippine Islands, Thailand, and Vietnam.
Black or African American	A person having origins in any of the black racial groups of Africa. Terms such as "Haitian" or "Negro" can be used in addition to "Black or African American."
Hispanic or Latino	A person of Cuban, Mexican, Puerto Rican, South or Central American, or other Spanish culture or origin, regardless of race. The term, "Spanish origin," can be used in addition to "Hispanic or Latino."
Native Hawaiian or Other Pacific Islander	A person having origins in any of the original peoples of Hawaii, Guam, Samoa, or other Pacific Islands.
White	A person having origins in any of the original peoples of Europe, the Middle East, or North Africa.

See https://obamawhitehouse.archives.gov/omb/fedreg 1997standards.

Table C Classification of diabetes.

Classification of diabetes	Definitions
T1DM	Study subjects were diagnosed with type 1 diabetes mellitus.
T2DM	Study subjects were diagnosed with type 2 diabetes mellitus.
GDM	Study subjects were diagnosed with gestational diabetes mellitus.
IGT	Study subjects were diagnosed with impaired glucose tolerance.
IFG	Study subjects were diagnosed with impaired fasting glucose.

T1DM, type 1 diabetes mellitus; T2DM, type 2 diabetes mellitus; GDM, gestational diabetes mellitus; IGT, impaired glucose tolerance; IFG, impaired fasting glucose; DM, diabetes mellitus

Table D Drug type and drug name for diabetes.

Drug type I	Drug type II	Drug name
oral antidiabetic agents (OADs)	Metformin	
	Xanthylureas/Sulfonylureas/Sulphonylureas	glipizide
		glybenclamide
		glyburide
	Glinides	Nateglinide
		Biguanides
	Thiazolidinediones	troglitazone
	α-glucosidase inhibitor	miglitol
	DPP-4 inhibitor	alogliptin
		yildagliptin
		sitagliptin
	Glucagon-like peptide-1 receptor agonist (GLP-1 RA)	
	Bile acid chelator	
	CYCLOSET (bromocriptine)	
	Sodium glucose cotransporter 2 inhibitor (SGLT2i)	
inject hypoglycemic drugs (IHDs)	Insulin/Insulin analgue	
	Fast-acting insulin analogs (Lispro, Aspart)	
	Short-acting insulin (Humulin-R, Actrapid-P)	
	Intermediate-acting insulin (Humulin-N, Protaphane)	Isophand Insulin(NPH insulin)
	Long-acting insulin (Protamine Zinc Insulin ,PZI)	
	Premixed insulin (Huminlin-70/30, Mixtard)	

Table E Diabetes technology

Classification of diabetes technology	Devices name		Description
self-monitoring of blood glucose (SMBG)	Continuous glucose monitoring (CGM) devices	Real-time CGM (rtCGM)	CGM systems that measure and display glucose levels continuously.
		Intermittently scanned CGM (isCGM)	CGM systems that measure glucose levels continuously but only display glucose values when swiped by a reader or a smartphone.
		Professional CGM	CGM devices that are placed on the patient in the provider's office (or with remote instruction) and worn for a discrete period of time (generally 7–14 days). Data may be blinded or visible to the person wearing the device. The data are used to assess glycemic patterns and trends. These devices are not fully owned by the patient—they are a clinic-based device, as opposed to the patient-owned rtCGM/isCGM devices.
Insulin Delivery		Insulin pumps	NA

American Diabetes Association; 7. Diabetes Technology: Standards of Medical Care in Diabetes—2021. Diabetes Care 1 January 2021; 44 (Supplement 1): S85–S99. *https://doi.org/10.2337/dc21-S007*

Table F Complications of diabetes

Type of complications	Classification of complications
Chronic	Diabetic cerebrovascular disease
	Diabetic neuropathy
	Diabetic peripheral neuropathy (DPN)
	Diabetic retinopathy (DR)
	Diabetic nephropathy (DN)
	Diabetic foot
	Infection
Acute	Diabetic ketoacidosis (DKA)
	Hyperosmolar hyperglycemic state (HHS)

Table G Health baseline information

Classification	Included variables/Options
Demographic characteristics	Age,Sex
Disease history and clinical symptoms	Clinical symptoms, Complications, History of disease, Medication history, Family history
Blood pressure	SBP, DBP, MAP
Energy intake	No fixed form
Mental health	No fixed form
Quality of life	No fixed form
Other risk factors	Such as sedentary behavior, smoking, drinking, and so on.

Table H Laboratory testing information

Classification	Included variables/Options
Blood lipid test	Adiponectin
	Apolipoprotein A1
	Apolipoprotein B
	Apolipoprotein B-48 (ApoB48)
	Apolipoprotein B/ apolipoprotein A1
	Fasting non-esterified fatty acids
	Free fatty acids
	HDL-C
	HDL/LDL ratio
	HDL2-C
	LDL-C
	LDL/HDL ratio
	Leptin
	Lipid accumulation product(LAP)
	Non-HDL-C
	Ox-HDL
	Ox-HDL/HDL-C
	Ox-LDL
	Ox-LDL/LDL-C
	Remnant-like lipoprotein particle cholesterol (RLP-C)
	Resistin
	S-cholesterol
	S-triglycerides
	TC
	TC/ApoB
	TC/HDL-C
	TC/HDL-C ratio
	TG
	Visfatin
	VLDL apoB absolute secretion rate
	VLDL apoB fractional catabolic rate
	VLDL apoB Pool size
	VLDL-C
	et. al
Diabetes laboratory test	CPI
	Fasting C-peptide
	FPG
	FPI
	Glucagon
	HbA1c
	HOMA2-IR
	HOMA-IR

	M/I<br>OGTT<br>Postprandial glucose<br>et. al
Inflammation indicators	CRP<br>hsCRP<br>IL-10<br>IL-18<br>IL-18/IL-10<br>IL-1β<br>IL-6<br>sTNF-a R1<br>TNF-α<br>et. al
Kidney function test	Albuminuria<br>BUN<br>Creatinine<br>GFR<br>UCN3<br>Urinary albumin-to-creatinine ratio<br>et. al
Cardiac function test	Ankle brachial index (ABI)<br>Aortic augmentation index<br>Aortic pulse wave velocity<br>Atrial fibrillation/flutter<br>Average heart rate<br>BNP<br>Cardiac index<br>Cardiac procedures/conditions<br>Cardio-ankle vascular index<br>Central pulse pressure<br>Diastolic dysfunction classification<br>Diastolic function<br>Ejection fraction<br>IVCT<br>IVRT<br>IVSD<br>IVST<br>LAVI<br>Left ventricular ejection fraction(LVEF)<br>Left ventricular hypertrophy<br>Left ventricular mass<br>Left ventricular RWT(relative wall thickness)<br>LVMI

	LVMI<br>MPI<br>Pulse wave velocity(PWV)<br>Restriction score<br>Stiffness Index(ASI)<br>et. al
Liver function test	Albumin<br>ALT<br>AST<br>AST/ALT ratio<br>FLI<br>FSI<br>GGT<br>et. al
Endothelial function testing	Ascular endothelial growth factor<br>Brachial artery diameter<br>Brachial PP(pulse pressure)<br>Endothelin-1<br>Endothelium independent vasodilation<br>Systemic vascular resistance<br>Systemic vascular resistance index (SVRI)<br>VEGFA receptor-1<br>VEGFA receptor-2<br>Vascular endothelial growth factor(VEGFA)<br>et. al
Routine blood test	Hemoglobin<br>Leucocytes<br>Lymphocytes<br>Monocytes<br>Neutrophil counts<br>Neutrophils<br>Platelets<br>Segmented neutrophil count<br>White blood cell<br>et. al
Thyroid function test	TSH, et. al
Sex hormone	Estradiol<br>Testosterone<br>Testosterone/SHBG<br>Urinary FSH<br>Urinary LH<br>et. al
Other laboratory tests	No fixed form

Table I Physical fitness evaluation

Classification	Included variables/Options
Assess current physical activity level	YES, NO
Participates in regular exercise	YES, NO
Body composition	BMI<br>Weight<br>Waist circumference<br>Height<br>Body fat percentage<br>Waist hip ratio(WHR)<br>Fat free mass (FFM)<br>Body fat mass<br>Hip circumference<br>Total body fat<br>et. al
Cardiorespiratory fitness	Heart rate<br>VO2peak/ $\dot{V}O^{2}_{peak}$<br>VO2max/ $\dot{V}O^{2}_{max}$<br>Maximal work load capacity (Wmax)<br>Wmax (maximal workload capacity)<br>RER<br>6-min walk distance<br>Resting energy expenditure<br>et. al
Muscular fitness	Range of Motion ROM(Ranges of Motion)<br>Lactate threshold<br>Lower body strength<br>Lower body Muscle strength<br>Upper body Muscle strength<br>Upper body strength<br>Knee extension force<br>Muscle fatigue(L) Extensors<br>Muscle fatigue(R) Extensors<br>Muscular work (N m)<br>Muscular work<br>Single repetition bench press<br>Single repetition lat pull down<br>1RM(one repetitive maximum) of LE(lower extremity)<br>1RM(one repetitive maximum) of UE(upper extremity)<br>muscle volume<br>1RM lower members<br>1RM total 6 exercises

	1RM upper members
	Average strength
	Knee extension
	Knee flexion
	Lactate
	Leg press
	M-value (mg/kg fat-free mass/min)
	Maximal strength (L) Extensors
	Maximal strength (L) Flexors
	Maximal strength (R) Extensors
	Maximal strength (R) Flexors
	Maximal voluntary contraction
	Muscle area (both legs)
	Muscle attenuation
	Muscular strength Chest press
	Muscular strength Leg press
	Muscular strength Row
	Single repetition maximum bench press
	Single repetition maximum lat pull down
	Total strength
	et. al

Table J The method of exercise risk evaluation

Contents of exercise risk evaluation	The method of exercise risk evaluation
Exercise preparticipation health screening	American College of Sports Medicine Preparticipation Screening (ACSMPS)
	The Physical Activity Readiness Questionnaire for everyone (PAR-Q+)
Exercise testing	Cardiorespiratory fitness (CRF)
Medical history and cardiovascular disease risk factor assessment	Interview/ laboratory assessment

Table K Classification of exercise

Classification of exercise	Function
Aerobic (cardiorespiratory endurance)	Aerobic exercise improves cardiorespiratory endurance.
Resistance	Resistance exercises can improve muscle fitness (strength, endurance and power).
Flexibility (stretching)	Joint range of motion (ROM) or flexibility can be improved across all age groups by engaging in flexibility exercises.
Neuromotor exercise	Neuromotor exercise training involves motor skills such as balance, coordination, gait, and agility and proprioceptive training and is sometimes called functional fitness training.
Combined	Any combination of two or more of the above.

Table L The special name of PA protocol

NO.	The special name of PA protocol
1	Activities of daily living (ADL) or leisure-time physical activity (LTPA) or self reported PA or daily regular exercise
2	High intensity intermittent training (HIIT)
3	Interval aerobic training (IAT)
4	Moderate continuous training (MICT)
5	High-intensity exercise(IHE)
6	Aerobic circuit training(ACT)
7	Continuous physical activity (CONT)
8	Sprint interval training (SIT)
9	Acute inspiratory muscle training(AIMT)
10	Continuous walking (CW)
11	Interval-walking(IW)
12	Progressive resistance training (PRT)
13	Acute high intensive training(AHIT)
14	Aerobic and resistance training (ATRT)
15	Arm swing exercise (ASE)
16	Low-intensity exercise training(LIE)
17	Maximal voluntary contraction (MVC)
18	Moderate-intensity aerobic exercise (MIAE)
19	Supervised exercise training (SET)
20	et. al

Table M The elements of physical activity protocol

NO.	Elements	Details
1	Classification	Aerobic (cardiorespiratory endurance), Resistance, Flexibility (stretching), Neuromotor exercise.
2	Frequency	Frequency, which refers to how often you exercise (i.e., the number of days/times per week dedicated to an exercise program).
3	Sets(n)	Refers to how many sets you take in resistance training.
4	Repetition per set (n)	Refers to the number of repetitions per set in resistance exercise.
5	Rest between sets(s)	Refers to the interval time between each set in resistance training.
6	Weight(RM)	Refers to how hard when you resistance exercise. The 1-RM, the greatest resistance that can be moved through the full range of motion (ROM) in a controlled manner with good posture. Percent of 1-RM or a multiple RM is a reliable indicator of muscle strength.
7	Intensity calculation indicator	Calculated metrics for estimating exercise intensity (%of heart rate reserve (HRR), maximal volume of oxygen consumed per unit time (VO2max), et. al)
8	Training intensity	Training intensity, which refers to how hard when you exercise.
9	Training intensity grade	Training intensity grade, which refers to the grade of how hard when you exercise.
10	Warm-up intensity	Warm-up intensity, which refers to how hard you warm-up before exercise.
11	Recovery intensity	Recovery intensity, which refers to how hard you recovery after exercise.
12	Time	Time, which refers to how long you exercise per time (min).
13	Type(mode)	Type, which refers to the mode or what kind you exercise.
14	Volume	Volume, which refers to the total amount of exercise per week or a period time.
15	Progress	Progress, which refers to increasing any of the components of the exercise principle of personalized exercise prescription as tolerated by the individual.
16	Warm-up/stretching/cool down	This part is also an integral part of the exercise prescription, which is a transitional phase that allows the body to adjust to the changing physiologic, biomechanical, and bioenergetic demands of the specific exercise session, that will be employed during exercise to help facilitate a more relaxed physiologic state.
17	Intertrial interval	Refers to the interval time of interval training protocols.
18	Duration	Refers to the duration of the training protocol.
19	Medical supervision	The implementation of the training protocol requires medical supervision by appropriately trained individuals.
20	Intensity monitor method	Refers to the means or measures taken in the process of implementing the exercise program to monitor the intensity of exercise.
21	Warning	Refers to special precautions before, during and after the implementation of the exercise protocol.
22	Contraindication	Refers to special groups of people who are not suitable for this exercise protocol.
23	Protocol	The description of exercise intervention regimens in the original literature.

Table N Intensity calculation indicator conclusion

Classification of exercise	Intensity calculation indicator	Training Intensity Grade				
		Very light	Light	Moderate	Vigorous	Near-maximal to maximal
Aerobic (cardiorespiratory endurance) Flexibility (stretching) Neuromotor exercise	%HRmax %HRpeak	<57	57-63	64-76	77-95	>=96
	%HRR	<30	30-39	40-59	60-89	>=90
	%VO2max %VO2peak	<37	37-45	46-63	64-90	>=91
	RER		<0.90		0.90-0.95	>1.0;1.2(Anaerobic exercise)
	RPE	<9	9-11	12-13	14-17	>=18
Resistance	%1-RM	<30	30-49	50-69	70-84	>=85

HRmax, maximal heart rate; HRpeak, peak heart rate; HRR, heart rate reserve; VO2max, maximal volume of oxygen consumed per unit time; VO2peak, peak volume of oxygen consumed per unit time; RER, respiratory exchange ratio; RPE, rating of perceived exertion;

Garber CE, Blissmer B, Deschenes MR, Franklin BA, Lamonte MJ, Lee IM, Nieman DC, Swain DP; American College of Sports Medicine. American College of Sports Medicine position stand. Quantity and quality of exercise for developing and maintaining cardiorespiratory, musculoskeletal, and neuromotor fitness in apparently healthy adults: guidance for prescribing exercise. Med Sci Sports Exerc. 2011 Jul;43(7):1334-59. doi: 10.1249/MSS.0b013e318213fefb. PMID: 21694556.

Table O The classification of PA's effect

NO.	Classification of PA's effect	Details
1	Better glycaemic control	Physical activity improves plasma glucose control, improves insulin sensitivity, such as lowering FPG, HbA1c, etc.
2	Improve physical fitness	Physical activity can improve body composition, aerobic capacity, resistance capacity, etc.
3	Better disease or complication prevention and control	Physical activity can prevent or delay the onset and progression of pre-diabetes or diabetes (complication).
4	Reduce cardiovascular risk factors	Physical activity can reduce cardiovascular risk factors (such as lowering blood lipids, controlling blood pressure, etc.).
5	Improve metabolism	Improve metabolism-related functions, such as improving lipid metabolism, improving energy metabolism, etc.
6	Promote cardiovascular benefits	Physical activity could improve cardiac and vascular function (such as improve central arterial stiffness, augmentation index, autonomic control function, etc.).
7	Improve inflammatory response	Physical activity improves inflammation.
8	Improve mental health	Physical activity can improve mental health (such as daily emotion affect, depressive symptoms, work-related stress, mental fatigue, distress etc.).
9	Improve health-related quality of life	Physical activity can improve health-related quality of life (especially for diabetes with complications.).
10	Improve gestational diabetes outcomes	Physical activity modifies adverse pregnancy outcomes in gestational diabetes.
11	Improve neural function	Physical activity can improve neural function (such as autonomic neural function etc.).
12	Improve immune function	Physical activity can improve immune function (such as inflammation and metabolic stress responses, inflammatory mediator etc.).
13	Improve cognitive function	Physical activity can improve congnitive function (especially for elderly.)

Table P The adverse events of PA

NO.	Classification of adverse events	Details
1	Prevent glucose metabolism disorders	Adverse events of glucose metabolism disorders caused by physical activity in diabetic patients, such as hypoglycemia events, hyperglycemia events, ketoacidosis events, etc.
2	Prevent cardiovascular negative events	The occurrence of cardiovascular negative events during exercise varies directly with the prevalence of diagnosed or occult CVD in the study population, such as AMI, ventricular fibrillation, etc.
3	Prevent musculoskeletal injury	Exercise induced musculoskeletal injuries such as fractures, sprains and strains, etc.
4	Prevent adverse respiratory events	Adverse respiratory events caused by exercise, such as diving sports barotrauma events, post-exercise dyspnea caused by a history of lung disease, etc.
5	Prevent environment induced negative events	Adverse exercise events caused by inappropriate temperature and humidity in the exercise environment, such as heat stress, hypothermia, etc.
6	Prevent renal dysfunction	Events of renal dysfunction such as exercise-induced abnormal microalbuminuria and albuminuria at rest.
